# ULTRA‐Metrics: Delphi‐Derived Framework for Assessing Ultrasound Competency

**DOI:** 10.1002/jum.70074

**Published:** 2025-10-07

**Authors:** Steve Reid, Alberto Goffi, Ean Tsou, Emanuele Pivetta, Suean Pascoe, Jessica Solis‐McCarthy, Mark Foster, Chris Gelabert, Mike Smith, Colin Bell, Erica Clarke Whalen, Hannah Latta, Janeve Desy, Simon Hayward, Hayley Israel, Andrew Leamon, Marcus Peck, Adrian Wong, Tanping Wong, Chris Yap, Emma M.L. Chung

**Affiliations:** ^1^ Executive Office Presuna Coleman Alberta Canada; ^2^ Interdepartmental Division of Critical Care Medicine University of Toronto Toronto Ontario Canada; ^3^ Innovation and Entrepreneurship University of Calgary Calgary Alberta Canada; ^4^ Division of Emergency Medicine and High Dependency Unit, Department of Medical Sciences University of Turin Turin Italy; ^5^ Executive Office Zedu Ultrasound Training Melbourne Victoria Australia; ^6^ Department of Emergency Medicine University of Texas Health San Antonio San Antonio Texas USA; ^7^ Department of Emergency Medicine, Department of Surgery Trauma Critical Care University of Texas Health San Antonio San Antonio Texas USA; ^8^ Department of Physiotherapy, College of Biomedical and Life Sciences Cardiff University Wales UK; ^9^ Department of Emergency Medicine University of Calgary Calgary Alberta Canada; ^10^ Department of Internal Medicine University of Manitoba Manitoba Canada; ^11^ Department of Paramedicine Auckland University of Technology, Manukau South Campus Auckland New Zealand; ^12^ Department of Critical Care Wellington Free Ambulance Wellington New Zealand; ^13^ Emergency Department Wellington Regional Hospital Wellington New Zealand; ^14^ Department of Medicine University of Calgary Calgary Alberta Canada; ^15^ Department of Physiotherapy Blackpool Teaching Hospitals NHS Foundation Trust Blackpool UK; ^16^ Division of Pulmonary and Critical Care University of New Mexico Albuquerque New Mexico USA; ^17^ Department of Emergency Medicine Presbyterian Santa Fe Medical Center Santa Fe New Mexico USA; ^18^ Department of Anaesthesia and Intensive Care Medicine Hampshire Hospitals NHS Foundation Trust Winchester UK; ^19^ Department of Critical Care King's College Hospital London UK; ^20^ Department of Medicine Weill Cornell Medicine New York USA; ^21^ Department of Emergency Sheffield Teaching Hospitals NHS Foundation Trust Sheffield UK; ^22^ Department of Women and Children's Health, School of Life Course and Population Sciences Faculty of Life Sciences and Medicine, King's College London London UK

**Keywords:** competency, metrics, POCUS, standards, training, ultrasound

## Abstract

**Objectives:**

Ultrasound competency is critical in modern healthcare, yet no standardized framework currently supports ultrasound skill monitoring across diverse clinical settings and user types. Existing frameworks often lack generalizability, overemphasize exam counts, and fail to assess key skills such as interpretation, limiting ultrasound's safe and effective integration into clinical practice. The objective of this study is to develop a consensus‐based, universal framework for monitoring ultrasound competency across clinical applications and disciplines.

**Methods:**

A modified Delphi process was conducted with an international panel of Point‐of‐Care ultrasound experts. Panelists independently evaluated framework elements categorized by competency domains (experience, skills, autonomy), skill domains (indication, acquisition, interpretation, clinical integration), metrics (eg, exam counts, entrustability, interpretation accuracy, etc.), answer sets (score‐based inputs used by assessors), and score criteria (requirements for each score). Consensus thresholds were defined as strong consensus at >84%, and weak consensus at 68–84%. Two Delphi rounds were completed.

**Results:**

Nineteen experts participated across 2 Delphi rounds. Strong consensus was reached to include 3 competency domains (experience, skills, autonomy) and 4 skill domains (indication, acquisition, interpretation, and clinical integration). Optional components, including the use of acquisition skill trees and varied answer set granularity, were favored by some participants to allow ultrasound programs to tailor the framework to specific examinations, assessment scenarios, and job roles.

**Conclusion:**

The resulting modular framework provides a flexible, consensus‐based approach to ultrasound competency assessment, enabling cross‐program comparisons and evaluation of training methods. Validation across diverse settings is needed to support its use in global competency standards and ultrasound education expansion.

**Abbreviations**
ACCORD: ACurate COnsensus Reporting Document
ACEP: American College of Emergency Physicians
AI: artificial intelligence
CDE: common data element
COPD: chronic obstructive pulmonary disease
EPA: entrustable professional activity
IRB: Institutional Review Board
MOES: Modified Ottawa Entrustability Score
OSCE: objective structured clinical examination
PGY 2: postgraduate year 2
POCUS: point‐of‐care ultrasound
UCAT: Ultrasound Competency Assessment Tool

The use of ultrasound in clinical practice requires skilled ultrasound practitioners to ensure accurate, efficient, and effective clinical decision making. Inadequately trained practitioners may miss pathology or reach an incorrect diagnosis, which can have devastating consequences for individual patients and reduce the overall quality of patient care.^1^ Standardization of ultrasound competency metrics could be an important step toward raising educational standards and increasing the effectiveness of patient care.^2^ Development of a common competency framework suited to diverse ultrasound training environments and disciplines presents several challenges:

1. The diverse backgrounds and clinical experience of ultrasound practitioners (various medical specialties, nurses, midwives, paramedics, physiotherapists, sonographers, radiographers);
2. Varying trajectories in acquiring practical skills and familiarization with ultrasound technology;
3. A wide range of ultrasound applications and examination types (eg, image‐guided procedures, point‐of‐care ultrasound, and diagnostic imaging and screening applications);
4. Varied scope of practice and levels of responsibility of practitioners.

Existing frameworks are typically categorized as either exam‐specific or exam‐agnostic. Exam‐agnostic frameworks, such as the I‐AIM framework^3^ and the Ultrasound Competency Assessment Tool (UCAT),^4^ offer structured approaches but have been found to be impractical to implement,^5^ demonstrate high inter‐rater variability,^6^ or do not assess competency holistically across experience, skills, and autonomy domains.^7^ Conversely, exam‐specific frameworks, such as skill trees and objective structured clinical examinations (OSCEs), focus narrowly on specific ultrasound skills or clinical scenarios, limiting generalizability.^7^ A recent study evaluating existing ultrasound competency frameworks, conducted by Israel et al (2023), highlighted that existing competency assessment methods are not sufficiently holistic, robust, or flexible enough for widespread use.^5^ Limitations include significant resource demands, complex data gathering processes, subjective scoring (with high inter‐rater variability), and assessments being insufficiently holistic (with key skills being overlooked).^6^, ^7^ Scoring methods for individual competency components, such as the Modified Ottawa Entrustability Scale^8^ (which rates trust in a learner's readiness to perform certain clinical tasks independently based on demonstrated competence) and the American College of Emergency Physicians (ACEP) Quality Assessment Scale^9^ (which rates ultrasound acquisition quality and completeness of the examination views), offer generalizable and accurate measurements but have not yet been integrated into a comprehensive competency framework.^10^ Some ultrasound software platforms incorporate tools to facilitate the monitoring of ultrasound trainees, simplifying implementation and data capture, but are not evidence‐based and face limitations related to generalizability, flexibility, and holistic assessment.^11^, ^12^, ^13^

The absence of a standardized, universally applicable ultrasound competency framework presents several challenges affecting ultrasound users and, ultimately, patient outcomes.

1. Significant variability exists across competency guidelines issued by professional medical societies and ultrasound programs internationally.^14^ Most current guidelines emphasize numeric exam‐count thresholds as a surrogate measure of competency, reflecting experience rather than directly measuring skill or autonomy.^7^ Consequently, learners who progress more slowly may achieve numeric targets without acquiring adequate skill or autonomy, potentially compromising ultrasound application in clinical settings and introducing patient safety risks.
2. Ultrasound training capacity is further constrained by limited availability of expert trainers.^15^ Despite improved access to ultrasound technology and structured curricula, significant expert mentorship and time investment remain necessary for trainees to achieve competency. Faster learners may require less supervision (Figure 1), potentially freeing expert resources; however, current competency standards typically allocate equal supervision and exam counts regardless of individual learning pace.^16^
3. Challenges in validating novel training innovations, such as virtual mentorship^17^ and artificial intelligence (AI) training tools,^18^ potentially hinder evidence‐based widespread adoption of training innovations. Promising innovations could enhance access to experts, expand training capacity, and accelerate trainee competency progression.^19^
4. Variability in competency metrics across ultrasound programs limits opportunities for robust benchmarking between programs and multicenter research.^20^

**Figure 1:**
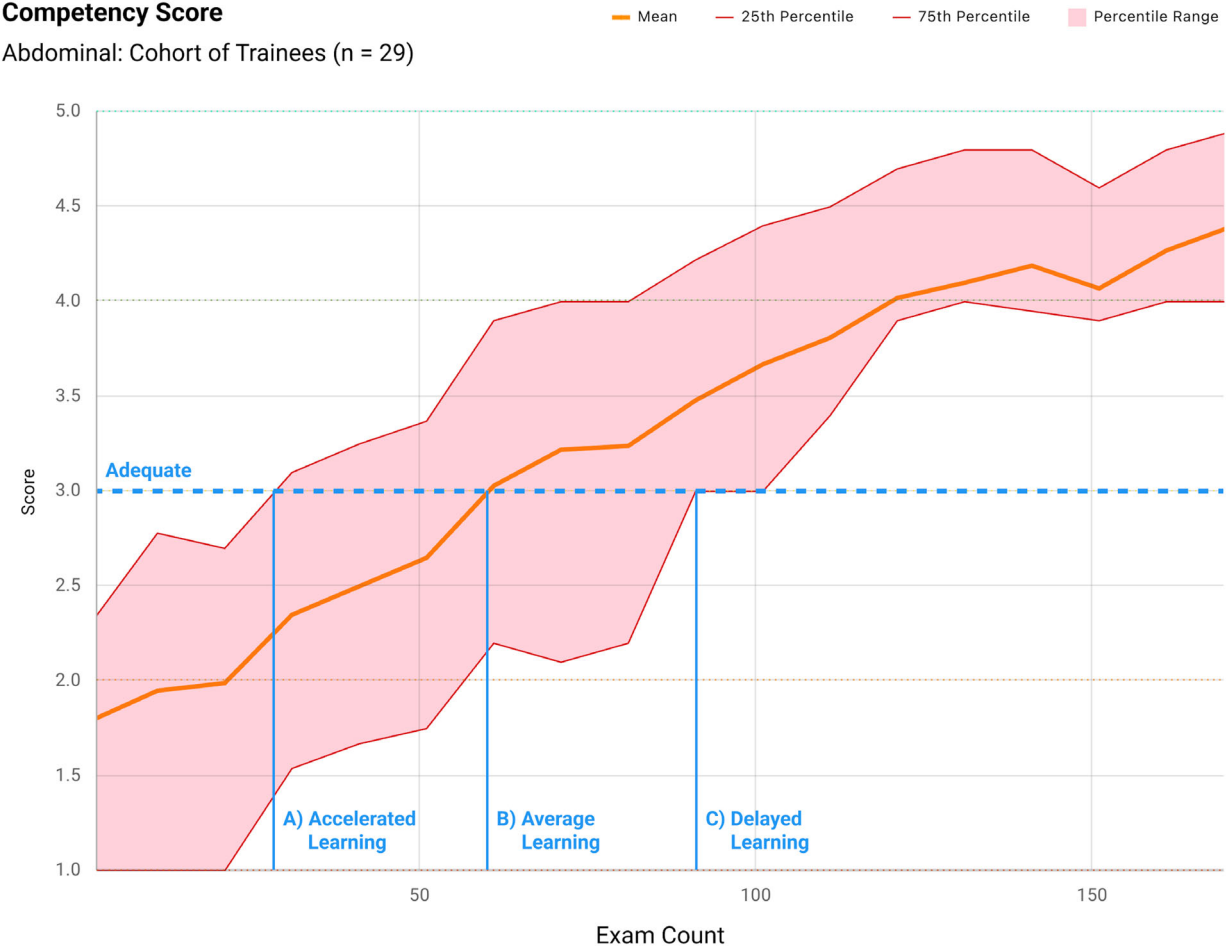
Competency curves. It is well known that the number of exams required to reach competency varies significantly between ultrasound trainees. The figure shows a learning trajectory for a group of ultrasound users as a function of the number of exams performed. The shaded regions represent the variability in learning rates within a group of ultrasound users. By applying a standardized approach, it becomes possible to evaluate how the number of examinations required to achieve proficiency varies across examination types and training programs.

This study aimed to establish a consensus‐based universal ultrasound competency framework—ULTRA‐metrics, with 4 primary design objectives:

1. Measure a trainee's competency holistically (beyond exam counts) with additional metrics to quantify experience, autonomy, and skills;
2. Ensure broad adaptability across all ultrasound applications, geographies, and training programs/disciplines through robust and generalizable measurements that are simple to capture;
3. Facilitate comparison of datasets through the development of framework common data elements (CDEs)^21^;
4. Ensure the framework is modular so it can evolve over time as new ultrasound competency metrics and training methods become available.

A consensus‐based approach to deriving this ULTRA‐metrics framework was chosen to maximize the chances of achieving these design objectives through harnessing the knowledge and experience of a diverse panel of experts.

## Materials and Methods

### Study Design and Setting

A modified Delphi method was used to achieve expert consensus on a first version (version 1.0) of the ULTRA‐metrics competency framework. The study was reported using the ACurate COnsensus Reporting Document (ACCORD) reporting guidelines.^22^ The process was conducted entirely online using web‐based questionnaires (Google Forms, Google LLC, Mountain View, CA) and video meetings (Google Meet) for group and 1–1 discussions. The Project Lead (SR) was responsible for designing the study as well as directing and coordinating the Delphi rounds. As this study does not involve patients, clinical trial registration was not required.

### Expert Panel and Recruitment

This study focused on experts with experience teaching point‐of‐care ultrasound (POCUS), whose skill set includes core diagnostic imaging competencies and extends to clinical integration and bedside decision‐making, supporting the relevance of findings to diagnostic imaging specialists. Expert panel members were recruited based on the following criteria: (1) a minimum of 3 years of ultrasound practice, and (2) at least 3 years of experience training others in ultrasound, including academic faculty, private course instructors, and global health educators, and (3) experience teaching POCUS. A total of 34 experts were emailed a detailed study overview, prioritizing experts with diverse specialty representation and geographic distribution. Potential participants were approached until a target minimum panel size of 20 experts was achieved.^23^

### Ethical Considerations

Institutional Review Board (IRB) ethics approval was obtained by the University of Turin, Italy, prior to starting the Delphi rounds (approval number 0426509). All members of the expert panel provided informed consent, which included freedom to withdraw from the study without negative consequences, and were also provided a detailed project overview. All data gathered from panel members during the Delphi process were pseudo‐anonymized using unique user codes managed exclusively by the Project Lead.

### Delphi Process and Analysis

The development of the framework followed 4 stages:

1. *Draft creation*: Literature review search of existing competency frameworks and scoring methods, along with expert panel contributions and completion of an optional questionnaire, were synthesized into an initial version of the framework and provided as background material to the expert panel.
2. *Draft refinement*: Group meetings with subgroups of the expert panel were conducted to further refine the framework and address key questions before launching the Delphi.
3. *Delphi*: Two rounds of anonymous online Delphi consensus‐building surveys, with individual responses, were conducted. Panel members were blinded to others' responses.
4. *Framework refinement*: Online consensus meetings and collaborative documents were used to finalize the framework.

The Project Lead and Delphi Analyst piloted the Delphi surveys internally prior to distribution to the expert panel to verify clarity and functionality of all questions and responses. No incentives were provided for participation. Each Delphi survey question was assigned a unique code to facilitate traceability. Score‐based questions were employed to determine consensus on framework items, while open‐ended questions enabled the expert panel to elaborate on responses and propose additional framework items. Framework items were classified into 4 categories to enhance the modularity of the Framework:

- *Domains*: Aspects of competency (eg, experience, autonomy, and skills).
- *Metrics*: Measurement of competency within a domain (eg, accuracy of interpretation within the skill domain).
- *Answer sets*: Scores an assessor uses to capture a metric (eg, scoring accuracy on a scale of 1–5).
- *Score criteria*: Criteria for the scores in each answer set (eg, accuracy score 1 means major inaccuracies; whereas an accuracy score of 5 means no findings were missed).

The domains were selected to align with the concept of entrustable professional activities (EPAs), where trainees are evaluated based on their ability to independently perform clinical tasks (experience and skills) without direct supervision (autonomy).^24^ Categories of metrics, answer sets, and score criteria were structured to align with existing ultrasound competency measures, such as the ACEP Quality Scale^9^ and the Modified Ottawa Entrustability Score.^8^

The primary outcome measure for each Delphi round was the agreeance score, which quantified consensus on framework items using the percentage of agreement method.^25^ Agreeance scores for competency domains and metrics were calculated using a proportion‐based method, with experts selecting “include,” “neutral,” or “exclude”; the agreeance score represents the percentage of “include” responses.^25^ Agreeance scores for answer sets and score criteria used a 5‐point Likert scale and weighted mean calculation converted to a percentage to measure central tendency.^26^ Consensus thresholds were defined as strong (>84%), weak (68–84%), and none (<68%).^27^ The 68% threshold was chosen to ensure that over two‐thirds of experts agreed, surpassing random chance and accommodating exploratory findings where moderate dissent is acceptable. The 84% threshold was set to approach near‐unanimity, minimizing outlier influence and ensuring robust conclusions. These thresholds align with findings from a systematic review, which reported a median consensus threshold of 75%, with variations depending on study objectives and desired stringency.^28^

After the first Delphi round, the Delphi Analyst compiled and analyzed data in collaboration with the Project Lead, producing anonymized quantitative summaries of aggregated agreeance scores reflecting group‐level consensus per question. These summaries were quality‐assured by the Project Lead and subsequently distributed to the expert panel prior to the second round. Experts were not permitted to revise their individual responses based on group feedback. All decisions regarding inclusion or exclusion of framework items were based exclusively on expert panel consensus. Framework items achieving strong consensus were excluded from the second round to make the process more efficient, while items with weak or no consensus were revised according to expert panel feedback. Additionally, new framework items suggested by experts through qualitative responses were incorporated into the subsequent Delphi round.

### Framework Refinement Process

To ensure consistency and comparability across ultrasound programs, all answer set scores were normalized to a 1–5 scale:

- *3‐point scales*: Mapped to 1, 3, and 5, preserving relative positioning (ie, score 1 → 1, score 2 → 3, score 3 → 5).
- *Binary scales*: Mapped to 1 and 3 (ie, score 1 → 1, score 2 → 3)

This mapping aimed to facilitate comparisons across different assessment formats, ensuring a score of 3 consistently reflects an “adequate” performance level.

At the conclusion of the Delphi rounds, framework items were classified into the following categories based on agreement scores:

- *Required*: Items achieving strong consensus (>84%), ensuring broad applicability across diverse programs.
- *Optional*: Items achieving weak consensus (68–84%), reflecting significant expert support but recognizing variability in applicability among programs.
- *Excluded*: Items achieving no consensus (<68%), indicating insufficient expert agreement for inclusion.

Finally, some optional metrics were classified as required based on satisfying both of the following rules:

- Metric's associated domain was also classified as required
- For a given domain, if no metrics reached strong consensus (>84%) the metric with the strongest weak consensus (68–84%) was classified as required

## Results

### Expert Panel

Of the 34 experts invited to join the expert panel, 23 (68%) agreed to participate. Non‐responses to invitations were followed up a maximum of 1 time. Of those who agreed, 16 (70%) participated in the initial draft creation stage, and 19 (83%) participated in the draft refinement stage. Two experts (9%) withdrew before commencing the Delphi stage, and 2 experts (9%) did not respond in either Delphi round. The final panel of experts who participated in the Delphi consensus process included 19 experts (Table 1).

**Table 1:** Expert Panel Characteristics

Characteristic	n	%
Years using ultrasound		
3–10	7	36.8
11–20	11	57.9
More than 20	1	5.3
Years of training others in ultrasound		
3–10	9	47.4
11–20	9	47.4
More than 20	1	5.3
Gender		
Male	11	57.9
Female	8	42.1
Primary specialty/discipline		
Emergency Medicine	7	36.8
Internal and Hospital Medicine	2	10.5
Critical Care Medicine	5	26.3
Physiotherapy	2	10.5
Ultrasound Medical Education	2	10.5
Pre‐Hospital Medicine	1	5.3
Geography of training^a^		
United States of America	6	31.6
Canada	4	21.1
United Kingdom	6	31.6
Italy	1	5.3
Australia	1	5.3
Jamaica	1	5.3
New Zealand	1	5.3

^a^ Experts can have experience training in multiple geographies.

### Delphi

The Delphi was conducted over 7 months between July 2024 and January 2025. In round 1, 18 experts participated, while 19 experts participated in round 2. At the beginning of round 1, experts reviewed an explanatory image and video describing the framework and were subsequently asked if they understood it. A total of 16 (88.9%) experts responded “yes,” 0 (0%) responded “no,” and 2 (11%) responded “mostly, but I have questions.”

Out of 138 items, 114 items reached consensus across both rounds of the Delphi. In round 1, 73 items were proposed, with 57 items achieving consensus for inclusion. Based on expert feedback in round 1, 11 items were modified and proposed in round 2, and 14 items were excluded. In round 2, 76 items were proposed, with 57 items achieving consensus for inclusion. The key results of the Delphi are detailed below. For a full list of the Delphi results of all 138 items, see Supporting Information S2.

#### [1] Global Competency

Initially, experts were presented with a 5‐point global competency score that integrated elements from the experience, autonomy, and skill domains into a single set of criteria. In round 1, there was no consensus on using a global competency score, although there was consensus in some of the score criteria. In round 2, the experts voted on 2 methods of capturing a competency score during assessments: (1) manual input with the 5‐point scale, (2) calculating based on entrustability and skill scores. No consensus was reached on either method; instead, experts' comments indicated a preference for measuring competency across 3 individual domains: experience, skills, and autonomy (Table 2).

**Table 2:** [1] Global Competency—Key Results

Framework Item	Consensus	Delphi Round	Question ID
**[Metric] Global Competency Score**: assessed with 5‐point scale	31.6% (none)	2	2:Q9d
**[Metric] Global Competency Score**: calculated based on Entrustability and Skill scores	67.4% (none)	2	2:Q9
**[Score Criteria] 5‐point scale**			
1: Requires close supervision for most of exam	85.6% (strong)	1	1:Q10b_1
2: Can complete parts of the examination independently under close supervision	83.3% (weak)	1	1:Q10b_2
3: Adequate practical skills. Can complete straightforward parts of the examination but needs close supervision for more challenging aspects or support with image interpretation	84.4% (strong)	1	1:Q10b_3
4: Good integration of practical skills and knowledge. Able to consistently complete both straightforward and more complicated parts of the examination with minimal supervisor intervention	83.3% (weak)	1	1:Q10b_4
5: Excellent integration of practical skills and knowledge. Able to consistently complete full scans to the standard of a qualified (first post) practitioner, including complicated aspects. Recognizes when a second opinion is required	85.6% (strong)	1	1:Q10b_5

#### [1.1] Experience (Competency Domain)

In round 1, 4 metrics were proposed. Exam counts and findings counts achieved consensus, while exam view counts and pathology counts did not achieve consensus (Table 3). No further items were proposed in round 2.

**Table 3:** [1.1] Experience Domain—Key Results

Framework Item	Consensus	Delphi Round	Question ID
**[Metric] Exam Counts**: number of exams performed of each type (ie, cardiac, lung, etc.)	94.4% (strong)	1	1:Q2a
**[Metric] Findings Counts**: number of findings interpreted by exam type (ie, lung B‐lines)	72.2% (weak)	1	1:Q4a
**[Metric] Exam View Counts**: number of specific views captured for each exam type (ie, cardiac PLAX, lung L1)	44.4% (none)	1	1:Q3a
**[Metric] Pathology Counts**: number of pathology interpreted for each exam type (ie, lung pneumonia)	66.7% (none)	1	1:Q5a

#### [1.2] Autonomy (Competency Domain)

In round 1, the metric of entrustability, trust in a learner's readiness to perform certain clinical tasks independently based on demonstrated competence, reached consensus (Table 4). The 5‐point score criteria to measure entrustability derived during the draft phase also reached consensus (Supporting Information S2). Experts recommended the score descriptors could be improved to better reflect supervisor involvement and learner independence, and some experts indicated the scale could be simplified to a 3‐point or binary scale. In round 2, the Modified Ottawa Entrustability Score (MOES) was proposed without and with single word descriptors (1 = dependent, 2 = guided, 3 = prompted, 4 = monitored, 5 = independent), and in 5‐point, 3‐point, and binary scale variants. The 5‐point MOES scale with single word descriptors achieved the strongest overall consensus, resulting in the exclusion of the 5‐point scale proposed in round 1 (Supporting Information S2). The 3‐point variant of the MOES scale also reached consensus; conversely, the binary variant did not reach consensus and was excluded (Supporting Information S2). The 5‐point MOES scale was designated as the default for the entrustability metric, as participants preferred this over a 3‐point scale (Table 4). No further domains within autonomy were evaluated.

**Table 4:** [1.2] Autonomy Domain—Key Results

Framework Item	Consensus	Delphi Round	Question ID
**[Metric] Entrustability:** trust in a learner's readiness to perform certain clinical tasks independently based on demonstrated competence	72.2% (weak)	1	1:Q9a
**[Answer Set: Default]** Modified Ottawa Entrustability Score: 5‐point scale	75.8% (weak)	2	2:Q8d_1
**1 (Dependent):** supervisor did it: trainee required complete guidance or was unprepared; supervisor had to do most of the work	81.1% (weak)	2	2:Q8b_1
**2 (Guided):** supervisor talked through it: trainee was able to perform some tasks but required repeated directions	83.2% (weak)	2	2:Q8b_2
**3 (Prompted):** supervisor needed to prompt: trainee demonstrated some independence and only required intermittent prompting	84.2% (strong)	2	2:Q8b_3
**4 (Monitored):** supervisor needed to be there just in case: trainee functioned fairly independently and only needed assistance with nuances or complex situations	84.2% (strong)	2	2:Q8b_4
**5 (Independent):** supervisor did not need to be there	90.5% (strong)	2	2:Q8b_5
**[Answer Set: Alternative]** Modified Ottawa Entrustability Score: 3‐point scale (scores 1, 3, 5 of 5‐point scale)	71.6% (weak)	2	2:Q8d_2

#### [1.3] Skill (Competency Domain)

In round 1, a single 5‐point global skill score metric was proposed, but did not reach consensus. Experts indicated a preference to measure skills through domains (indication, acquisition, interpretation, clinical integration). In round 2, an aggregated single skill score calculated from the skill domains was proposed, but did not achieve consensus (Table 5).

**Table 5:** Skill Domain—Key Results

Framework Item	Consensus	Delphi Round	Question ID
**[Metric] Global Skill Score:** assessed with 5‐point scale (1 = poor, 2 = fair, 3 = adequate, 4 = good, 5 = ideal)	61.1% (none)	1	1:Q8c
**[Metric] Aggregated Skill Score:** calculated using an aggregation of the individual Skill Domain scores	63.2% (none)	2	2:Q7b
Score threshold: <3 (not adequate) = not ready for clinical usage	74.7% (weak)	2	2:Q7d_1
Score threshold: ≥3 (adequate) = ready for clinical usage	75.8% (weak)	2	2:Q7d_2
**[1.3.1] Indication**	72.2% (weak)	1	1:Q12a
**[Metric] Appropriateness:** is the ultrasound exam chosen appropriate considering the patient's symptoms and medical history?	66.7% (none)	1	1:Q12b
**[Metric] Reasoning:** can the practitioner articulate a focused clinical question that ultrasound can address for this patient?	89.5% (strong)	2	2:Q3a
Preference of Reasoning over Appropriateness	84.2% (strong)	2	2:Q3c
**[Answer Set: Default] binary scale**	81.1% (weak)	2	2:Q3h_2
**1 (Poor):** does not provide a relevant clinical question for ultrasound evaluation, or the question is unclear or lacks specificity	70.5% (weak)	2	2:Q3g_1
**3 (Adequate):** relevant clinical question formulated	75.8% (weak)	2	2:Q3g_2
**[Answer Set: Alternative] 3‐point scale**	71.6% (weak)	2	2:Q3h_1
**1 (Poor):** no relevant clinical question formulated	76.8% (weak)	2	2:Q3e_1
**3 (Adequate):** relevant clinical question formulated, but it lacks specificity to the patient's condition	73.7% (weak)	2	2:Q3e_2
**5 (Ideal):** relevant clinical question formulated, and is specific to the patient's condition	76.8% (weak)	2	2:Q3e_3
**[1.3.2] Acquisition**	100% (strong)	1	1:Q13a
**[Metric] Quality + Completeness:** were the acquired ultrasound scans of enough completeness and quality to answer the clinical question?	83.3% (weak)	1	1:Q13b
**Metric capture options:** Quality and Completeness asked together or separately	74.7% (weak)	2	2:Q4
**[Capture Option 1] Quality + Completeness together**			
**[Answer Set]** ACEP 5‐point scale			
**1 (Poor):** no recognizable structures	83.2% (weak)	2	2:Q4d_1
**2 (Fair):** minimally recognizable structures but insufficient for diagnosis	82.1% (weak)	2	2:Q4d_2
**3 (Adequate):** minimal criteria met for diagnosis, recognizable structures but with some technical or other flaws	84.2% (strong)	2	2:Q4d_3
**4 (Good):** minimal criteria met for diagnosis, all structures imaged well	80.0% (weak)	2	2:Q4d_4
**5 (Ideal):** minimal criteria met for diagnosis, all structures imaged with excellent image quality	81.1% (weak)	2	2:Q4d_5
**[Capture Option 2] Quality + Completeness separate**			
**[Answer Set] Completeness 3‐point scale**			
**1 (Poor):** one or more required scan views missing	80% (weak)	2	2:Q4g_1
**3 (Adequate):** required scan views are complete, one or more optional scan views missing	70.5% (weak)	2	2:Q4g_2
**5 (Ideal):** required and optional scan views are complete	73.7% (weak)	2	2:Q4g_3
**[Answer Set] Quality 3‐point scale**			
**1 (Poor):** scans do not allow for accurate interpretation	82.1% (weak)	2	2:Q4j_1
**3 (Adequate):** scans allow for accurate interpretation, but scan quality could be improved	81.1% (weak)	2	2:Q4j_2
**5 (Ideal):** scans allow for accurate interpretation with excellent diagnostic scan quality	78.9% (weak)	2	2:Q4j_3
**[1.3.3] Interpretation**	100% (strong)	1	1:Q14a
**[Metric] Accuracy:** were relevant findings and pathology accurately interpreted?	100% (strong)	1	1:Q14b
**[Answer Set]** 3‐point scale	86.3% (strong)	2	2:Q5d_2
**1 (Poor):** Major inaccuracies	87.4% (strong)	2	2:Q5c_1
**3 (Adequate):** Minor inaccuracies only	87.4% (strong)	2	2:Q5c_2
**5 (Ideal):** No minor or major inaccuracies	87.4% (strong)	2	2:Q5c_3
**[1.3.4] Clinical Integration**	77.8% (weak)	1	1:Q15a
**[Metric] Effectiveness:** were ultrasound findings effectively integrated into clinical decision‐making and patient management?	72.2% (weak)	1	1:Q15b
**[Metric] Appropriateness:** were ultrasound findings appropriately integrated into clinical decision‐making?	73.7% (weak)	2	2:Q6a
Preference of appropriateness over effectiveness	78.9% (weak)	2	2:Q6c
**[Answer Set: Default]** 3‐point scale	75.8% (weak)	2	2:Q6h_1
**1 (Poor):** fails to appropriately apply ultrasound findings to clinical decisions	77.9% (weak)	2	2:Q6e_1
**3 (Adequate):** applies ultrasound findings appropriately but may overlook subtle details or additional relevant information	75.8% (weak)	2	2:Q6e_2
**5 (Ideal):** appropriately incorporates all ultrasound findings into clinical decisions without errors or omissions	78.9% (weak)	2	2:Q6e_3
**[Answer Set: Alternative]** binary scale	71.6% (weak)	2	2:Q6h_2
**1 (Poor):** fails to appropriately apply ultrasound findings to clinical decisions	76.8% (weak)	2	2:Q6g_1
**3 (Adequate):** applies ultrasound findings appropriately	77.9% (weak)	2	2:Q6g_2

The 4 skill domains (indication, acquisition, interpretation, and clinical integration) reached consensus for inclusion (Table 5). A common 5‐point scale was proposed (1 = poor, 2 = fair, 3 = adequate, 4 = good, 5 = ideal) for scoring the skill domains, with the criteria for each score being specific to each skill domain, and experts reached consensus that a score of 3 or greater indicates readiness for clinical usage (Table 5).

#### [1.3.1] Indication (Skill Domain)

*The indication skill domain refers to the ultrasound practitioner's ability to assess whether an ultrasound scan (or referral for a specific ultrasound examination) is appropriate to the patient's medical history, clinical symptoms*, *and any complementary tests or imaging. The ultrasound practitioner needs to be able to formulate a clear clinical question to answer with ultrasound*.

In round 1, weak consensus was reached to include indication as a skill domain. An appropriateness metric (ie, is the ultrasound exam chosen appropriate considering the patient's symptoms and medical history?) was proposed but did not reach consensus. Based on experts' feedback, a reasoning metric (ie, assessing whether the practitioner can articulate a focused clinical question) was introduced in round 2; this reached consensus. Binary and 3‐point scales were both proposed for reasoning, with consensus achieved for both, and a stronger expert preference for the binary scale (Table 5).

#### [1.3.2] Acquisition (Skill Domain)

*The acquisition skill domain refers to* the *skills of acquiring and optimizing ultrasound images*.

In round 1, there was unanimous consensus (100%) to include acquisition as a skill domain. Mentor‐assessed metrics of quality and completeness were proposed (ie, were all required views successfully obtained and of sufficiently high image quality to answer the clinical question?). The acquisition skill domain achieved consensus for inclusion (Table 5). While a 5‐point scale derived in the draft phase reached consensus, expert feedback from round 1 revealed differing preferences (Supporting Information S2). Some participants preferred assessing acquisition quality and completeness together (as a combined statement) using the 5‐point American College of Emergency Physicians (ACEP) scale^9^ (Table 5), while others preferred image quality and exam completeness to be presented as 2 separate questions. In round 2, experts reached a consensus to include the options of choosing either the ACEP 5‐point scale or separate quality and completeness questions with 3‐point scales (Table 5). The 5‐point scale proposed in round 1 was excluded from the framework as the ACEP 5‐point scale reached a higher consensus (Supporting Information S2).

#### [1.3.3] Interpretation (Skill Domain)

*The image interpretation skill domain refers to the practitioner's ability to distinguish normal from abnormal findings*.

In round 1 there was unanimous consensus (100%) to include both an interpretation skill domain and an accuracy metric (ie, were relevant findings and pathology accurately interpreted?) (Table 5). While a 5‐point scale derived from the draft phase reached consensus, expert feedback from round 1 indicated that the criteria for scores 3 (multiple minor inaccuracies—no major inaccuracies) and 4 (single minor inaccuracy—no major inaccuracies) were too similar (Supporting Information S2). This informed the introduction, in round 2, of a revised 5‐point scale and a simpler 3‐point scale. In addition, a confusion matrix (ie, true positive, true negative, false positive, false negative) to score the accuracy metric was included in round 2. In round 2, the revised 5‐point scale did not reach consensus on all of its score criteria and was excluded (Supporting Information S2). The 3‐point scale reached strong consensus while the confusion matrix reached weak consensus. Expert feedback from round 2 indicated that the confusion matrix is too cumbersome to use and does not support interpretation of scenarios with multiple findings; thus, it was excluded from the framework (Table 5).

#### [1.3.4] Clinical Integration (Skill Domain)

*The clinical integration skill domain refers to the practitioner's skill in integrating interpreted ultrasound findings into clinical management*.

In round 1, experts reached a weak consensus supporting the inclusion of a clinical integration skill domain. An effectiveness metric (ie, were ultrasound findings effectively integrated into clinical decision‐making and patient management?) reached consensus; however, expert feedback indicated that clinical effectiveness would be impractical to measure as this requires clinical follow‐up of subsequent patient outcomes. An alternative metric of appropriateness was therefore proposed (ie, were ultrasound findings *appropriately* integrated into clinical decision‐making?), which was proposed in round 2 and reached consensus. Expert feedback from round 1 also indicated a 5‐point scale was too granular, resulting in a 3‐point scale and a binary scale for the appropriateness metric being proposed in round 2, with experts preferring a 3‐point scale for measuring the appropriateness of clinical integration (Table 5).

#### [1.3.2.1] Acquisition Skill Tree

An acquisition skill tree is designed to enable granular assessment of the micro‐skills required for successfully performing ultrasound image acquisition. The skills tree summarized below was introduced in round 1; this comprises 11 micro‐skills with 4 possible answer sets to score each micro‐skill. Consensus was reached on 10 micro‐skills and 2 answer sets (Table 6), with no additions proposed in Round 2.

**Table 6:** [1.3.2.1] Acquisition Skill Tree—All Results

Framework Item	Consensus	Delphi Round	Question ID
**Patient positioning:** aligning the patient for imaging access	72.2% (weak)	1	1:Q16a_1
**Probe selection:** choosing the ultrasound probe for the exam	88.9% (strong)	1	1:Q16a_2
**Device preset:** pre‐configured settings for specific exam types	83.3% (weak)	1	1:Q16a_3
**Device modality:** ultrasound technique applied, for example, B‐mode, Doppler	72.2% (weak)	1	1:Q16a_4
**Device depth:** setting for ultrasound wave penetration depth	88.9% (strong)	1	1:Q16a_5
**Device gain:** adjustment of image brightness	88.9% (strong)	1	1:Q16a_6
**Labeling:** annotating images with identifiers or findings	66.7% (none)	1	1:Q16a_7
**Measurements:** quantifying anatomical sizes or pathologies on images	72.2% (weak)	1	1:Q16a_8
**Probe orientation:** aligning the probe with anatomical directions	77.8% (weak)	1	1:Q16a_9
**Probe control:** handling the probe for optimal image capture	77.8% (weak)	1	1:Q16a_10
**Landmarks:** recognizable features used for navigation and interpretation.	72.2% (weak)	1	1:Q16a_11
**[Answer set]** ^a^ 3‐point quality scale (1 = poor, 3 = adequate, 5 = ideal).	77.8% (weak)	1	1:Q16c_1
**[Answer set]** ^a^ 5‐point quality scale (1 = poor, 2 = fair, 3 = adequate, 4 = good, 5 = ideal).	56.9% (none)	1	1:Q16c_2
**[Answer set]** ^a^ Binary scale (error, correct)	44.4% (none)	1	1:Q16c_3
**[Answer set]** ^b^ Feedback in the answers: feedback on how to improve the micro skill within the answer—for example, Gain (too dark, too bright, adequate)	79.2% (weak)	1	1:Q16c_4

^a^ Same score criteria for answer set is used for all acquisition micro‐skills.

^b^ Different score criteria for answer set used for all acquisition micro‐skills. Defining the score criteria for “feedback in the answers” was out of scope for this study.

#### [2] Metadata

Metadata describes the context of the ultrasound assessment. All 5 metadata items proposed in round 1 achieved consensus. Experts proposed 9 additional metadata items during round 1, of which only 4 achieved consensus in round 2 (Table 7).

**Table 7:** [2] Metadata—All Results

Framework Item	Agreeance Score	Delphi Round	Question ID
**Exam type:** type of exam performed (ie, cardiac, lung, etc.)	94.4% (strong)	1	1:Q11a_1
**User:** who acquired and/or interpreted the ultrasound exam	83.3% (weak)	1	1:Q11a_2
**Acquisition user:** who acquired the ultrasound scans	89.5% (strong)	2	2:Q1a
**Interpretation user:** who interpreted the ultrasound scans	84.2% (strong)	2	2:Q1b
**Patient difficulty:** challenges performing the ultrasound exam due to patient circumstance	88.9% (strong)	1	1:Q11a_4
**Supervised vs unsupervised:** whether or not the exam performed under supervision	94.4% (strong)	1	1:Q11a_5
**Clinical vs training:** if the exam was performed clinically or for training purposes	94.4% (strong)	1	1:Q11a_6
**Cohort:** which group or cohort the user is associated with	89.5% (strong)	2	2:Q1c
**Acquisition setting:** ICU, ED, outpatient, etc.	78.9% (weak)	2	2:Q1g
**Ultrasound transducer type:** phased, linear, curved linear, etc.	57.9% (none)	2	2:Q1d
**Ultrasound device manufacturer:** company manufacturer	15.8% (none)	2	2:Q1e
**Ultrasound device model:** specific model from the manufacturer	10.5% (none)	2	2:Q1f
**Acquisition date:** specific date the ultrasound was performed on	57.9% (none)	2	2:Q1h
**Acquisition time:** specific time ultrasound was performed at	42.1% (none)	2	2:Q1i

### Framework Refinement

Framework refinement was conducted over a 2‐month period from January and February 2025. Based on expert panel feedback, the Project Lead implemented the following changes to the framework:

1. The acquisition skill tree was made optional. Although 3 micro‐skills reached strong consensus, experts emphasized the need to minimize data entry requirements to maximize adoption of the framework in busy clinical and training settings.
2. For metadata items, “user” was categorized as required, while “acquisition user” and “interpretation user” were categorized as optional to minimize data entry requirements as typically the same user is acquiring and interpreting.
3. For metrics with multiple answer sets, weak‐consensus answer sets were excluded if at least 1 alternative answer set reached strong consensus. This criterion applied only to the interpretation accuracy metric, resulting in the exclusion of the confusion matrix (weak consensus) in favor of the 3‐point scale (strong consensus).
4. Default options were established for metric and answer sets with multiple capture methods. Defaults were selected based on the highest achieved consensus.

**Figure 2:**
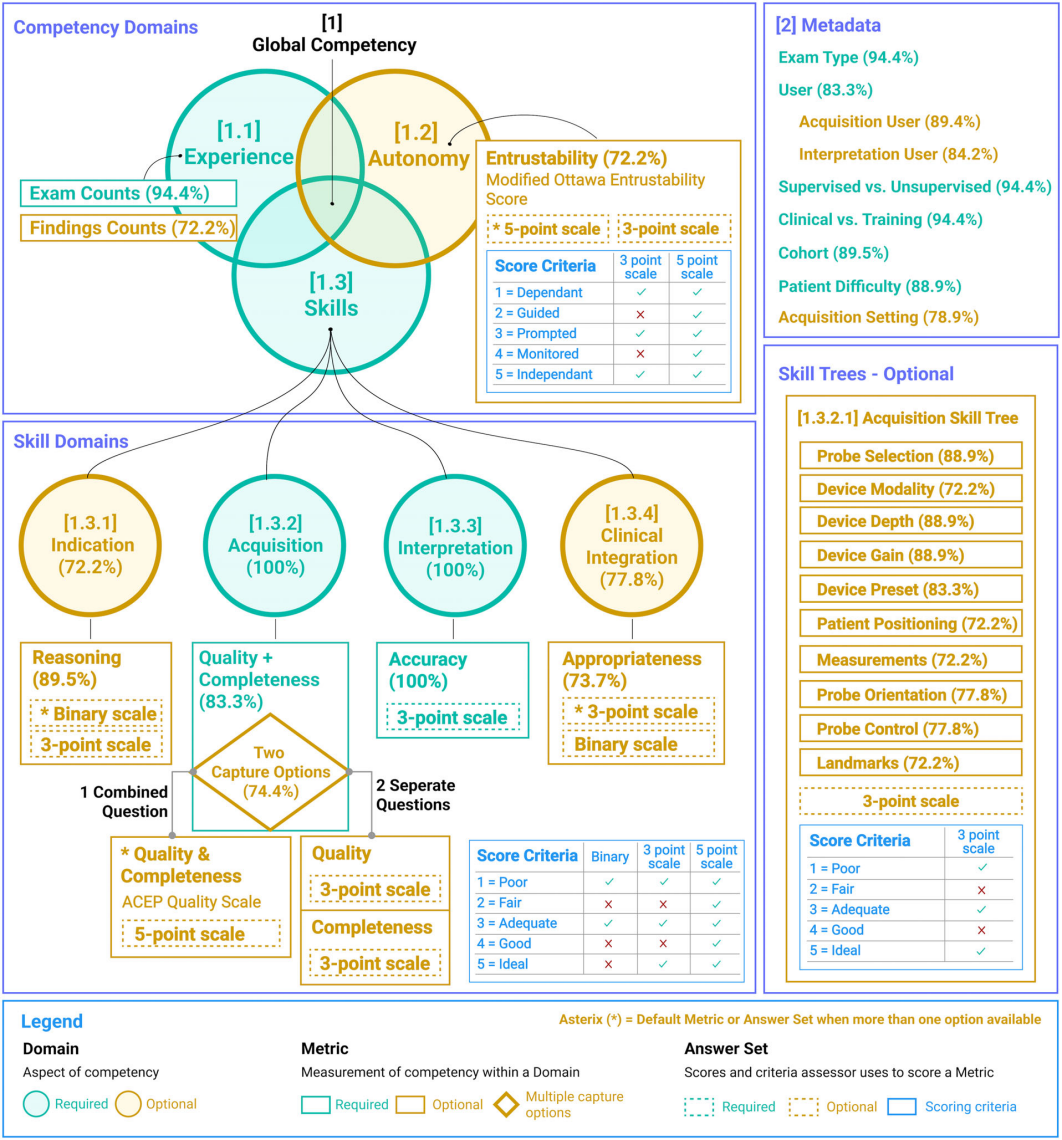
ULTRA‐metrics competency framework. Global Competency [1] is broken down into competency domains of experience [1.1], autonomy [1.2], and skills [1.3]. Skills are further broken down into skill domains of indication [1.3.1], acquisition [1.3.2], interpretation [1.3.3], and clinical integration [1.3.4]. During a competency assessment, metrics are captured using answer sets and score criteria to measure each competency and skill domain. The acquisition skill tree [1.3.2.1] is optional for more granular assessment of acquisition micro‐skills. [2] Metadata is also captured with each competency assessment to provide context behind the assessment. Required components are shown in green, while optional components are in yellow. Consensus agreement scores are indicated in brackets within the metric and skill domains. For metrics with multiple capture options and multiple answer sets, an asterisk (*) indicates the option that reached the highest consensus, which is used as the default option.

The final version of the ULTRA‐metrics competency framework is presented in Figure 2. After the framework refinement stage, 3 metrics and 6 metadata items were categorized as required, while 3 metrics, 3 metadata items, and the acquisition skill tree consisting of 10 micro‐skills were categorized as optional (Figure 2).

## Discussion

This Delphi study resulted in a consensus‐driven framework for measuring competency in ultrasound. This ULTRA‐metrics framework assesses competency in 3 key domains: experience, autonomy, and skills. Experience is assessed through a required exam count metric. Autonomy is evaluated using an optional entrustability metric (ie, how much supervision was required). Skills are assessed across 2 required skill domains: acquisition and interpretation, and 2 optional skill domains: indication and clinical integration. An acquisition skill tree with 10 micro‐skills was included as optional for more granular assessment of the acquisition skill domain. Six required metadata items were included: exam type, user, cohort, supervised vs. unsupervised, clinical vs. training, and patient difficulty. Three optional metadata items were included: acquisition user, interpretation user, and acquisition setting.

The ULTRA‐metrics framework addresses known limitations of existing frameworks, such as an over‐reliance on exam counts, lack of generalizability, and scoring subjectivity. This framework offers a modular, holistic approach to competency measurement with an emphasis on objective and easy‐to‐understand scoring criteria. It addresses the challenge of adaptability across ultrasound programs by incorporating both required and optional metrics, making it applicable to a wide range of ultrasound applications and user groups. It can be used with in‐person and unsupervised assessments with minimal required data input (3 metrics and 6 metadata items to be assessed by an external mentor) (Supporting Information S1). The ULTRA‐metrics framework can be adapted to suit various ultrasound programs and medical specialty needs through adding optional metrics and/or an acquisition skill tree that both allow for more holistic and granular competency measurement. The 2 optional skill domains, indication and clinical integration, allow flexibility to the user's scope of practice, as some medical specialties (eg, sonography) don't involve indication or clinical integration with ultrasound usage. The 3 different granularity options (5‐point, 3‐point, binary scales) for capturing metrics further enhance adaptability to individual program needs. The framework's utilization of CDEs facilitates consistent data collection across ultrasound programs, enabling robust multi‐program analyses. Additionally, consensus on 9 metadata items supports meaningful cross‐program dataset comparisons and aggregation.

To illustrate the application of the framework in a clinical scenario, consider a lung ultrasound performed by a postgraduate year 2 (PGY 2) trainee for a 65‐year‐old male with a past medical history of coronary artery disease, previous admission for heart failure, and chronic obstructive pulmonary disease (COPD) presenting with acute dyspnea after a fall. The trainee formulated a focused clinical question—“Does this patient have a pneumothorax, given recent trauma, or pulmonary edema or COPD exacerbation?”—meeting the reasoning metric (QID: 2:Q3a) within the indication domain (QID: 1:Q12a) and scored as Ideal using the 3‐point scale (QID: 2:Qe_3). All required views were obtained using an 8‐zone lung protocol, with minimal criteria met for diagnosis and all pleural and lung structures imaged well—Acquisition (QID: 1:Q13a) was therefore scored as 4 out of 5, indicating good image quality (QID: 2:Q4d_4). Interpretation (QID: 1:Q14a) identified bilateral lung sliding and diffuse B lines with right‐sided pleural effusion and no significant consolidations except at the right costophrenic angle; only a small left‐sided effusion was missed. This corresponded to an adequate score—minor inaccuracies only (QID: 2:Q5c_2)—for interpretation accuracy (QID: 1:Q14b). Clinical integration (QID: 1:Q15a) was appropriate (QID: 2:Q6a), with findings used to guide management with noninvasive ventilation and intravenous diuretics. The trainee required intermittent prompting, corresponding to a score of 3 out of 5 on entrustability (QID: 1:Q9a; 2:Q8b_3). Required metadata (Table 7) were captured alongside the assessment, including exam type (lung), user ID, trainee cohort (PGY 2), supervision status (supervised), clinical context (clinical use), and patient difficulty (yes, due to body habitus). This example demonstrates how the framework captures competency across domains while preserving contextual detail for meaningful interpretation. In this case, the trainee achieved a score of at least 3 in all assessed skill domains, indicating adequate competency; however, the entrustability score of 3 suggests that ongoing supervision remains necessary.

The ULTRA‐metrics framework demonstrates alignment with 2 existing ultrasound competency frameworks, I‐AIM^3^ and UCAT,^4^ while addressing their noted limitations. The I‐AIM framework is a structured model to guide ultrasound use through 4 steps: indication, acquisition, interpretation, and medical decision making.^3^ ULTRA‐metrics' skill domains mirror the I‐AIM steps and expand upon I‐AIM by incorporating experience and autonomy as core competency domains, enabling a more holistic assessment of ultrasound competency. Unlike I‐AIM, which offers a conceptual structure, ULTRA‐metrics provides clearly defined metrics with objective score criteria to support practical implementation. The UCAT framework assesses ultrasound competency across 4 skill domains: preparation, image acquisition, image optimization, and clinical integration; each rated on a 3‐point scale, alongside a global entrustment score.^4^ Comparing ULTRA‐metrics to UCAT, both frameworks share a foundation in competency‐based medical education and use entrustment scoring based on the Modified Ottawa Entrustability Score. UCAT assesses multiple anchors (behavior criteria) within each skill domain using a generalized 3‐point scale with score criteria: (1) competent performance of some criteria, (2) competent performance of most criteria, (3) competent performance of all criteria. For example, in the image acquisition skill domain, assessors use the single 3‐point scale to assess performance across 6 distinct anchors (positioning, probe selection, appropriate clinical indication, initial device settings, ensuring a clean transducer). In contrast, ULTRA‐metrics isolates a single metric within each skill domain (eg, reasoning in the indication skill domain), accompanied by answer sets and score criteria tailored to each metric to enhance clarity and objectivity, potentially overcoming the reported inter‐rater variability limitation of the UCAT framework. Furthermore, UCAT lacks a distinct skill domain for interpretation; instead, interpretation is grouped as 1 of 6 anchors within the broader clinical integration domain, making it difficult to isolate and assess interpretation skills independently. ULTRA‐metrics addresses this by evaluating interpretation as a standalone skill domain. Comparing derivation methodologies, I‐AIM was conceptually developed using educational and clinical quality improvement literature but did not undergo formal consensus methodology. In contrast, UCAT was derived using a modified Delphi process with a consensus threshold of ≥65% for item inclusion. This is similar to ULTRA‐metrics' weak consensus threshold (>68%); however, the introduction of a strong consensus threshold (>84%) in ULTRA‐metrics provides greater clarity in identifying more universally accepted items. The panel of experts involved in deriving the UCAT were all part of the Canadian Association of Emergency Physicians Emergency Ultrasound Committee (EUC), whereas ULTRA‐metrics' panel of experts had additional diversity in geography and medical specialties.

ULTRA‐metrics' autonomy domain, measured through entrustability, reflects the principles of EPAs,^24^ while the experience domain, measured by exam counts, aligns with existing professional ultrasound competency guidelines.^7^ Incorporating the Modified Ottawa Entrustability Score^8^ and the ACEP scale^9^ illustrates alignment with existing ultrasound competency scoring methodologies; however, expert feedback identified important limitations. Some experts outside of North America expressed concerns about the ACEP scale's inability to distinguish scenarios of high‐quality but incomplete image acquisitions (and vice‐versa), whereas North American experts generally found the ACEP quality scale sufficiently robust. Addressing this discrepancy, the ULTRA‐metrics framework enables the measurement of acquisition quality and completeness both as an aggregate single item and as 2 separate items for program flexibility. Additionally, consensus favored adding single‐word descriptors to the Modified Ottawa Entrustability Score, with experts expressing how it makes scoring more intuitive. This indicates that summarizing long score criteria with a single word could also help address inter‐rater variability limitations within existing competency frameworks.

A unique feature of ULTRA‐metrics compared to existing frameworks is the ability to correlate exam counts with any metric (eg, entrustability, acquisition quality, and completeness) to better indicate how many exams are required to reach certain competency thresholds (eg, readiness for clinical usage) across different cohorts and exam types, as well as potentially enabling more personalized competency pathways (Supporting Information S1). This has the potential to help address variability across competency guidelines issued by various professional medical societies and ultrasound programs internationally,^14^ increase expert capacity,^16^ and accelerate the adoption of ultrasound innovations such as virtual mentorship^17^ and AI.^18^

Notably, the expert panel did not reach consensus on utilizing a global competency score. Although criteria for a 5‐point scale achieved consensus, experts expressed concerns regarding accuracy and subjectivity using a global competency score, emphasizing the importance of distinct measurements across experience, autonomy, and skill domains. Similarly, the expert panel did not reach consensus on a global skill score and highlighted the need to measure competencies within individual skill domains separately. While a global competency score and skill score could reduce data input requirements, these findings underscore concerns of accuracy and subjectivity regarding oversimplification when evaluating nuanced ultrasound competencies.

Expert feedback regarding the exclusion of the clinical integration effectiveness metric highlighted the importance of selecting measures that are feasible to evaluate within the immediate context of an ultrasound encounter. Although the metric reached consensus for inclusion, its reliance on follow‐up of downstream patient interventions was viewed as impractical for routine assessment.

Additionally, expert panel feedback from round 1 emphasized reducing answer set granularity where possible. Experts favored binary and 3‐point scales over 5‐point scales for indication, interpretation, and clinical integration domains. However, a 5‐point scale was preferred for entrustability, suggesting that increased granularity is valuable for certain aspects of competency.

## Strengths and Limitations of the Delphi Process

A key strength of the Delphi process was the strong expert panel engagement across 2 Delphi rounds, with 86% and 90% participation, respectively. The iterative nature of the Delphi process and a high consensus threshold for inclusion of required framework items allowed items to be refined systematically. Moreover, broad geographical and specialty representation, although skewed toward emergency and critical care, enhanced the consensus quality and should support the framework's broader applicability. Nonetheless, the under‐representation of some specialties may limit certain specialty insights needed (eg, obstetrics, gynecology, vascular ultrasound, and echocardiography). The expert panel consisted of POCUS users, of which 16 (84%) were medically qualified doctors, providing robust applicability within POCUS programs but potentially limiting generalizability to diagnostic medical sonography and radiology settings. Future iterations should aim to diversify the expert panel to enhance validity and the broader applicability of the framework.

## Next Steps and Future Research

Moving forward, data collection and validation within training and clinical environments are essential to assess the framework's usability, robustness, and utility. Such efforts could generate new data confirming the number of examinations required to achieve defined skills thresholds. This may accelerate trainee pathways to competency, inform more precise competency standards, quantify the impact of training innovations (eg, virtual mentorship, AI), and enhance overall ultrasound training capacity through making better use of existing mentors.

While the framework is designed to meet current training needs of the clinical ultrasound community, its modular design will allow the framework to be updated over time to address emerging developments.

## Conclusion

This Delphi study resulted in a consensus‐derived ultrasound competency framework designed to measure competency holistically, beyond exam counts, and generalizable across exam types, geographic regions, ultrasound programs, and user groups. Strong consensus was achieved on a core set of measures that is feasible to implement in busy clinical and educational environments, with optional components offering flexibility to accommodate varying training needs and resource capacities.

With predefined consensus thresholds ensuring methodological rigor, the framework is ready for validation and data collection within training and clinical environments. It holds the potential to generate longitudinal competency curves that may accelerate trainee progression, inform more accurate competency standards, quantify the impact of educational innovations, and support the expansion of ultrasound training capacity globally.

## Supporting information


**Supporting Information S1.** Framework implementation.

**Supporting Information S2** All Delphi results.

## Data Availability

The data that support the findings of this study are available from the corresponding author upon reasonable request.
