# Supplementary material for: ULTRA‐Metrics: Delphi‐Derived Framework for Assessing Ultrasound Competency
Source: J Ultrasound Med. 2025 Oct 7;45(2):383–400. doi: 10.1002/jum.70074 (PMC12757764; doi:10.1002/jum.70074)
Supplement: Supplementary file 1 — Supporting Information S1. Framework implementation. [file JUM-45-383-s002.docx]

# Supplementary File A. Framework Implementation

**Figure 3.** In-person assessment workflow


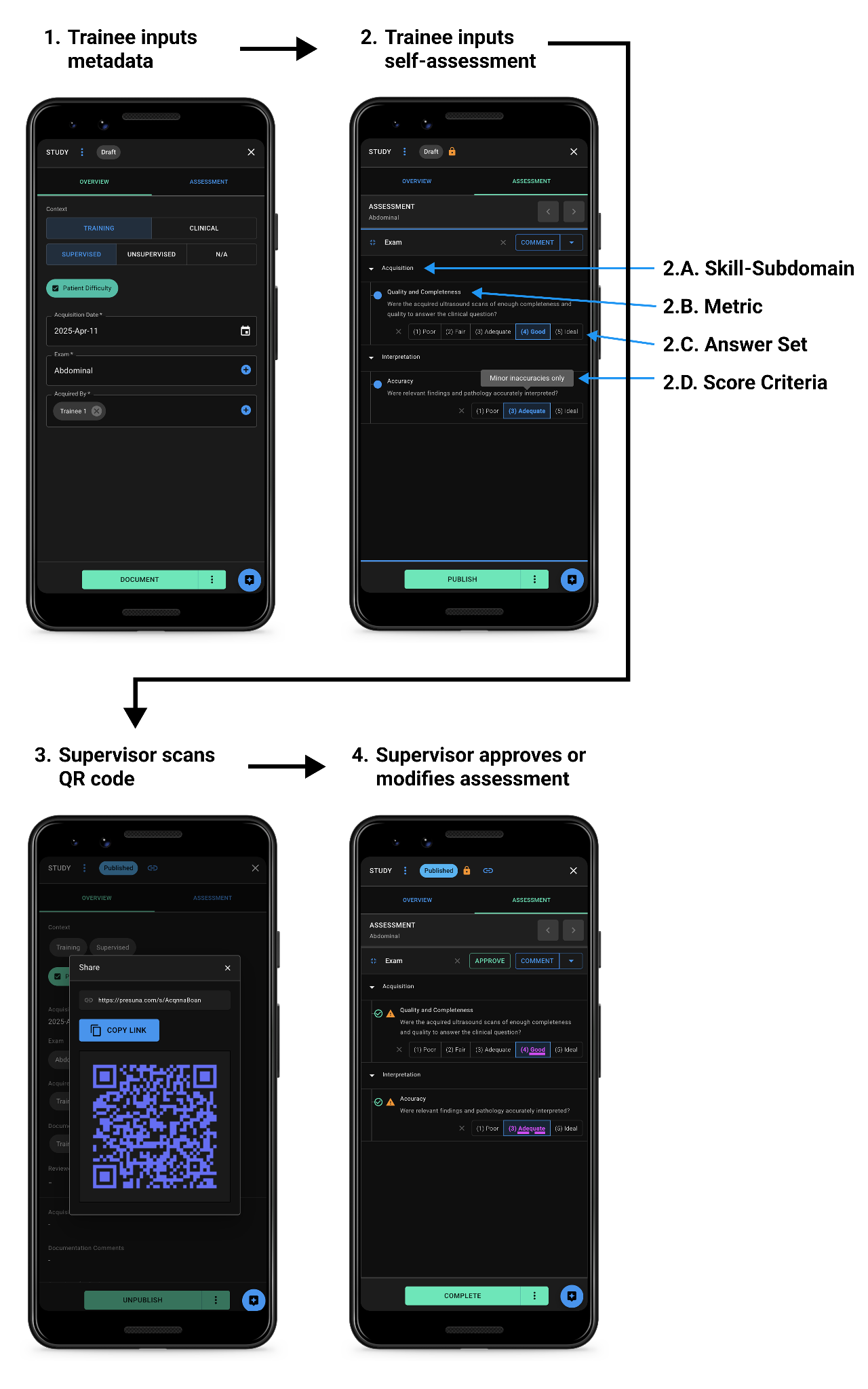


**Legend**

During an in-person assessment, ULTRA-metrics data can be captured using a mobile app. (1) A trainee opens the app on their mobile device, and inputs metadata describing their current ultrasound exam. (2) Trainee performs self-assessment using ULTRA-metrics (2.A) Skill-subdomains (2.B) Metrics (2.C) Answer Sets (2.D) Score Criteria. (3) Upon completion, trainee presents QR code for the supervisor. The supervisor scans the QR code with their mobile device camera and the self-assessment performed by the trainee is loaded on their mobile device’s web browser (no user account or app required). (4) The supervisor presses “approve” if they agree with the trainee’s self-assessment, and can modify any answers as required. The supervisor presses “complete” and the trainee’s competency curves are automatically updated.

**Figure 4.** Competency curve - acquisition quality and completeness


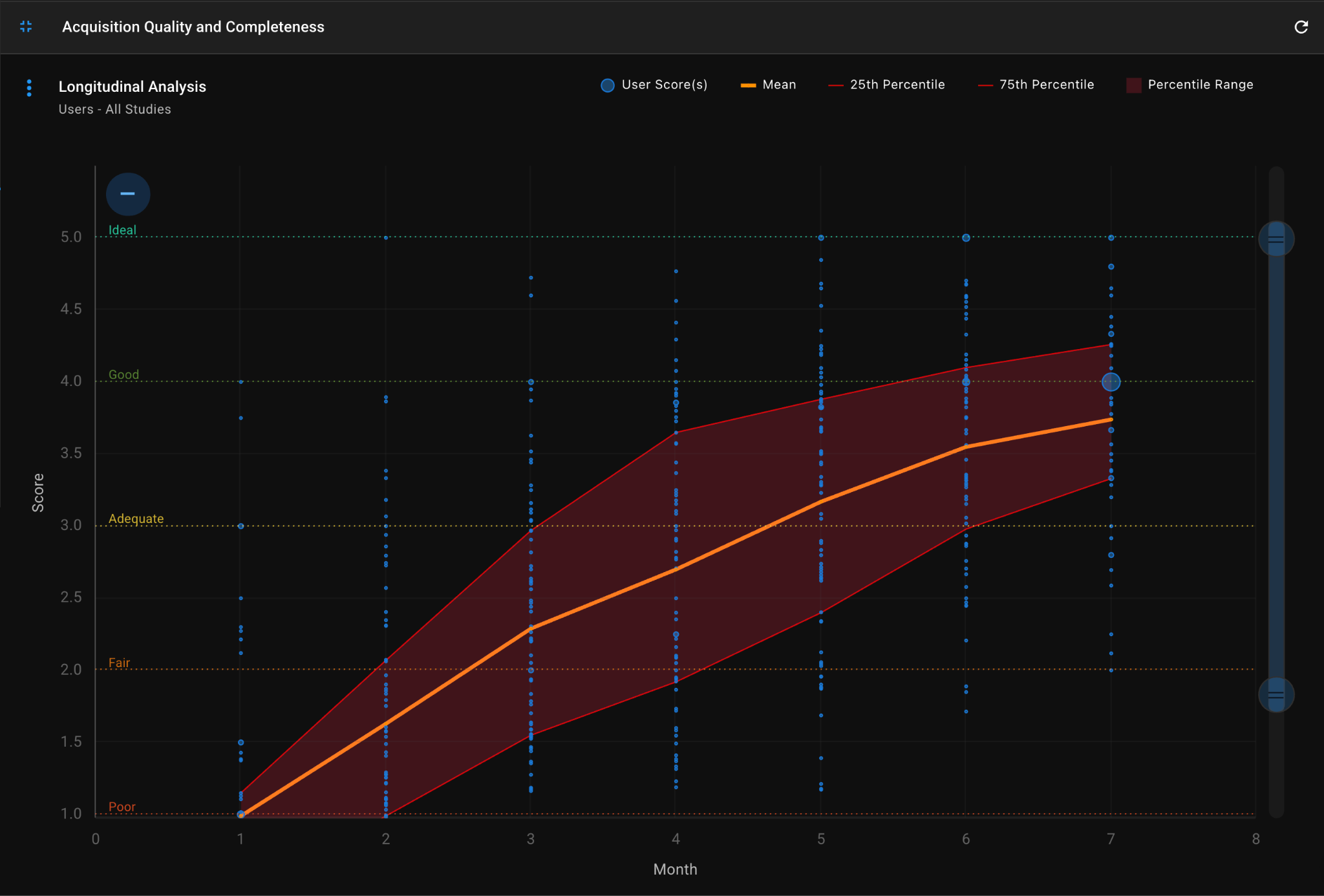


**Legend**

Longitudinal competency curve for a cohort of ultrasound users. This competency curve is for the acquisition quality and completeness metric and contains ULTRA-metrics data captured for every in-person and unsupervised assessment across the cohort.
