# Supplementary material for: ULTRA‐Metrics: Delphi‐Derived Framework for Assessing Ultrasound Competency
Source: J Ultrasound Med. 2025 Oct 7;45(2):383–400. doi: 10.1002/jum.70074 (PMC12757764; doi:10.1002/jum.70074)
Supplement: Supplementary file 2 — Supporting Information S2 All Delphi results. [file JUM-45-383-s001.docx]

# Supplementary File B. All Delphi Results

**Figure S3**. Competency score - Delphi result


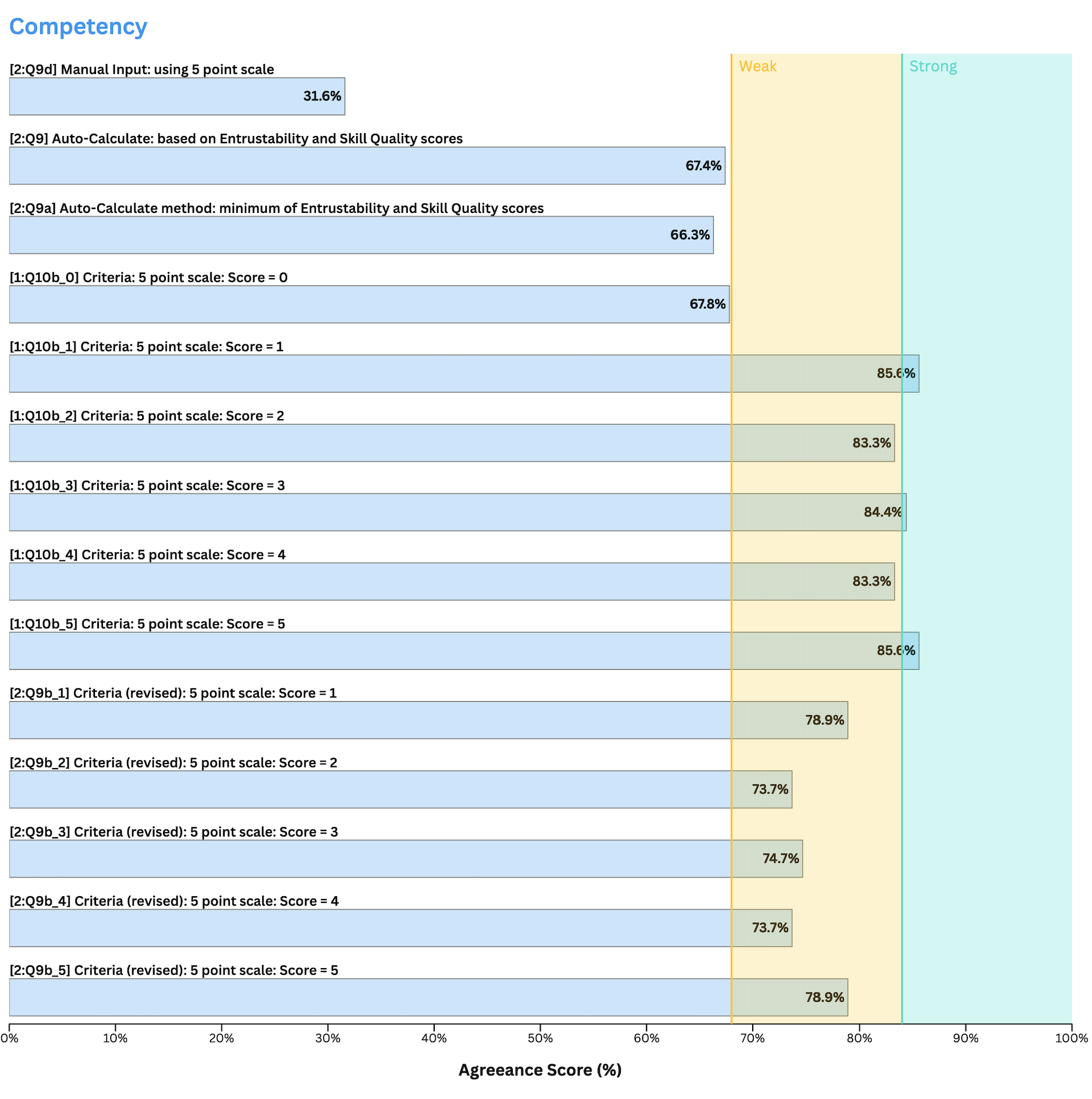


**Figure S4**. Competency domain: experience - Delphi result


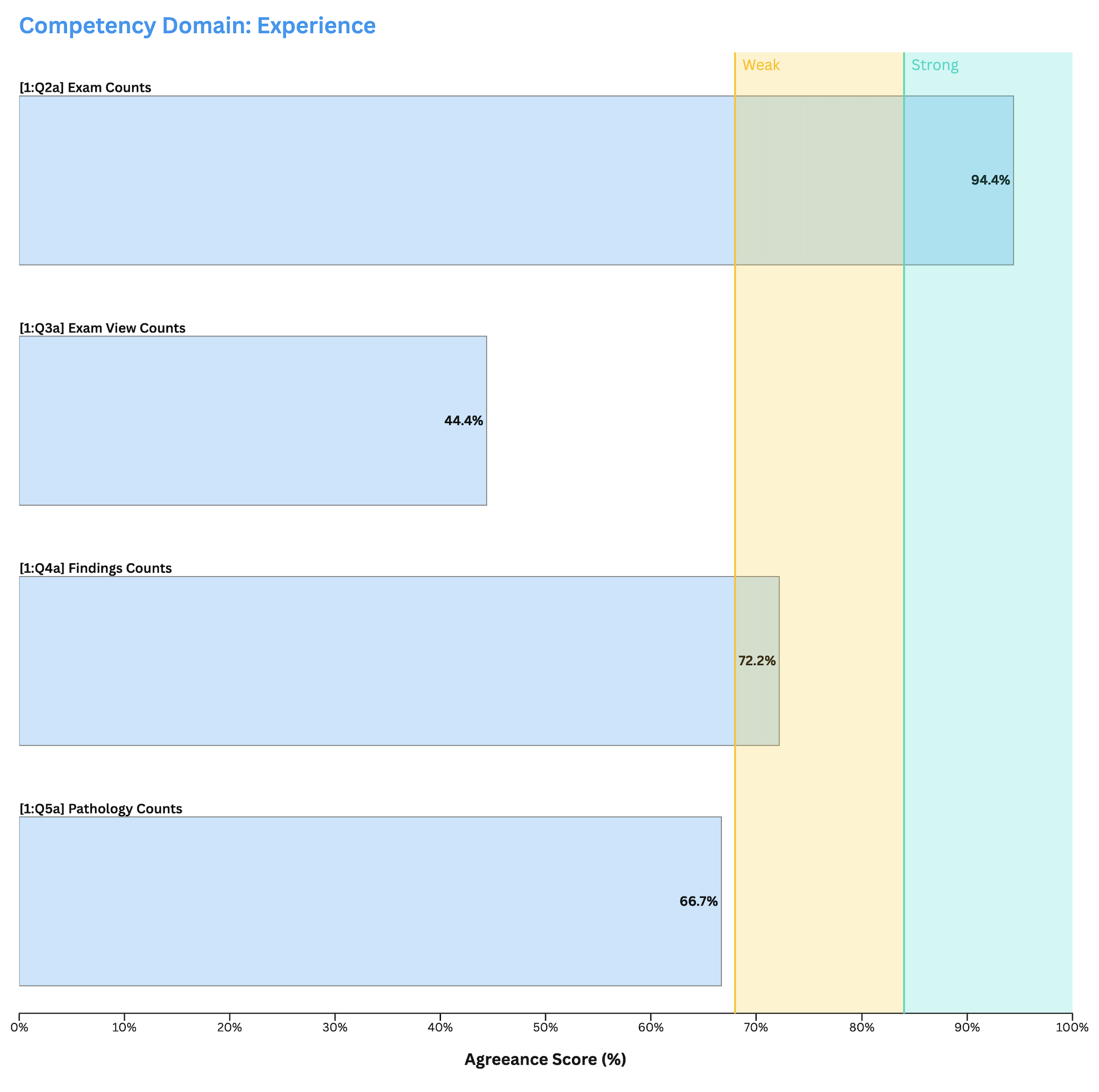


**Figure S5**. Competency domain: autonomy - entrustability metric - Delphi result


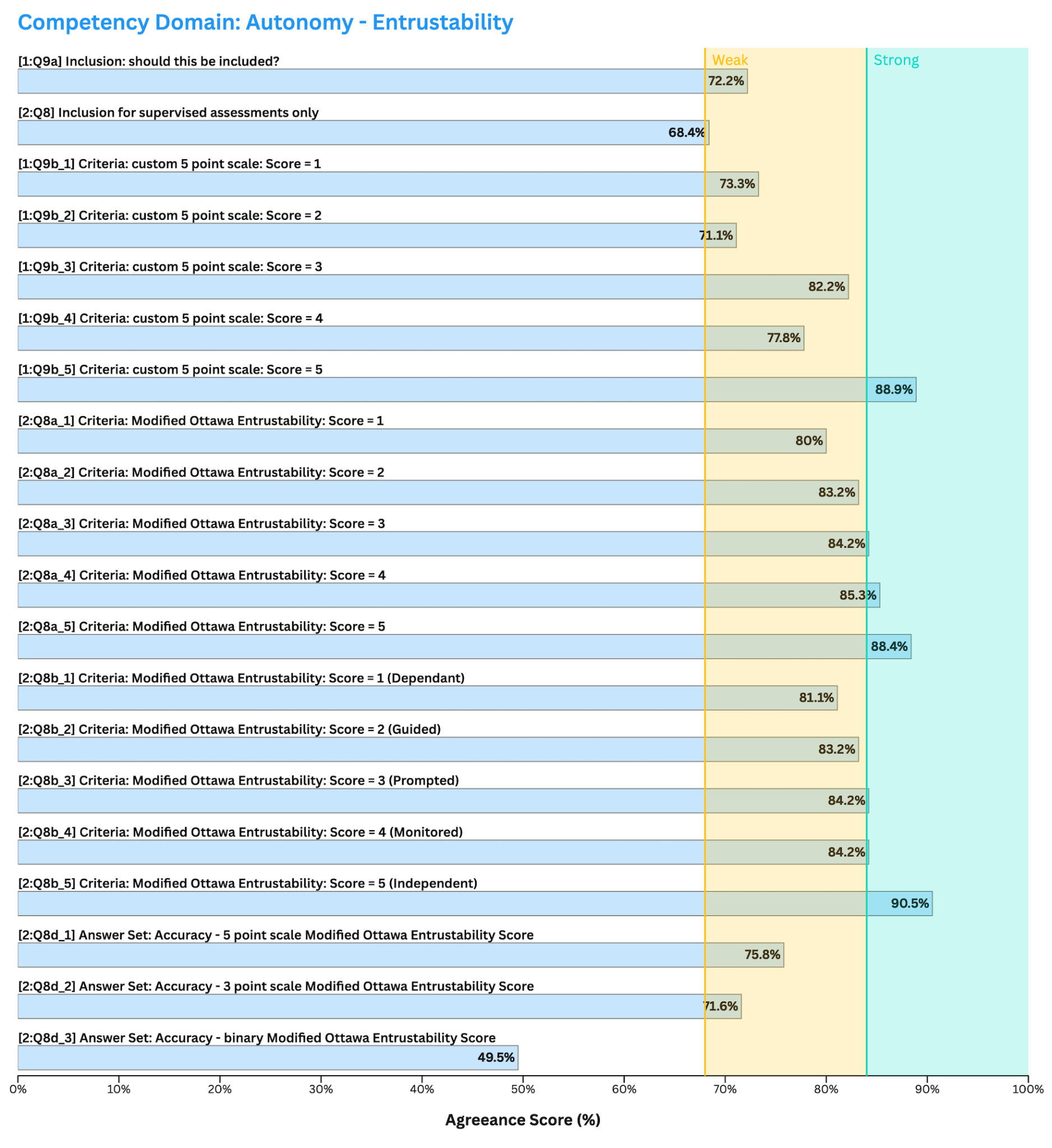


**Figure S6**. Competency domain: skills - Delphi result


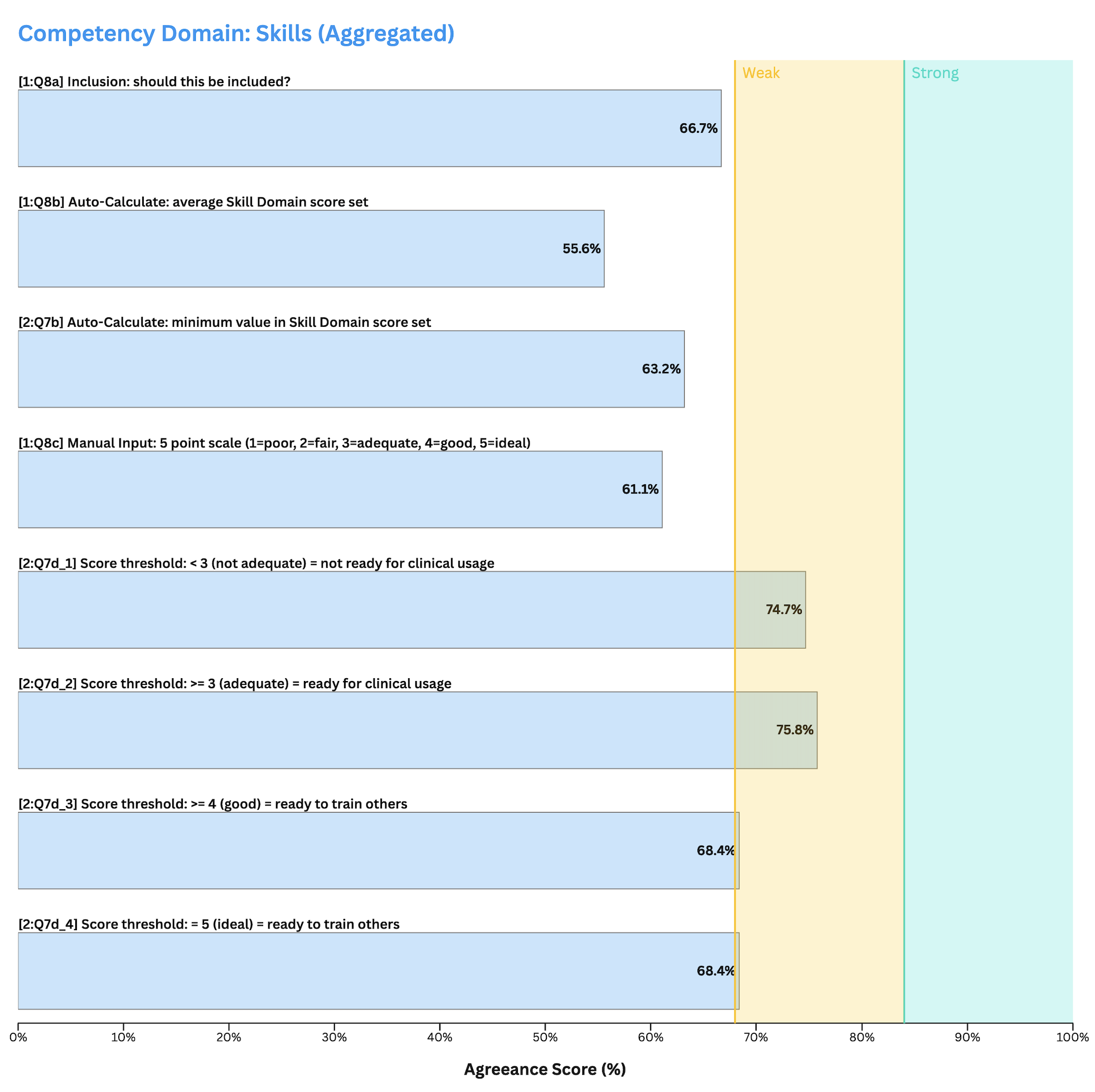


**Figure S7**. Skill domain: indication - Delphi result


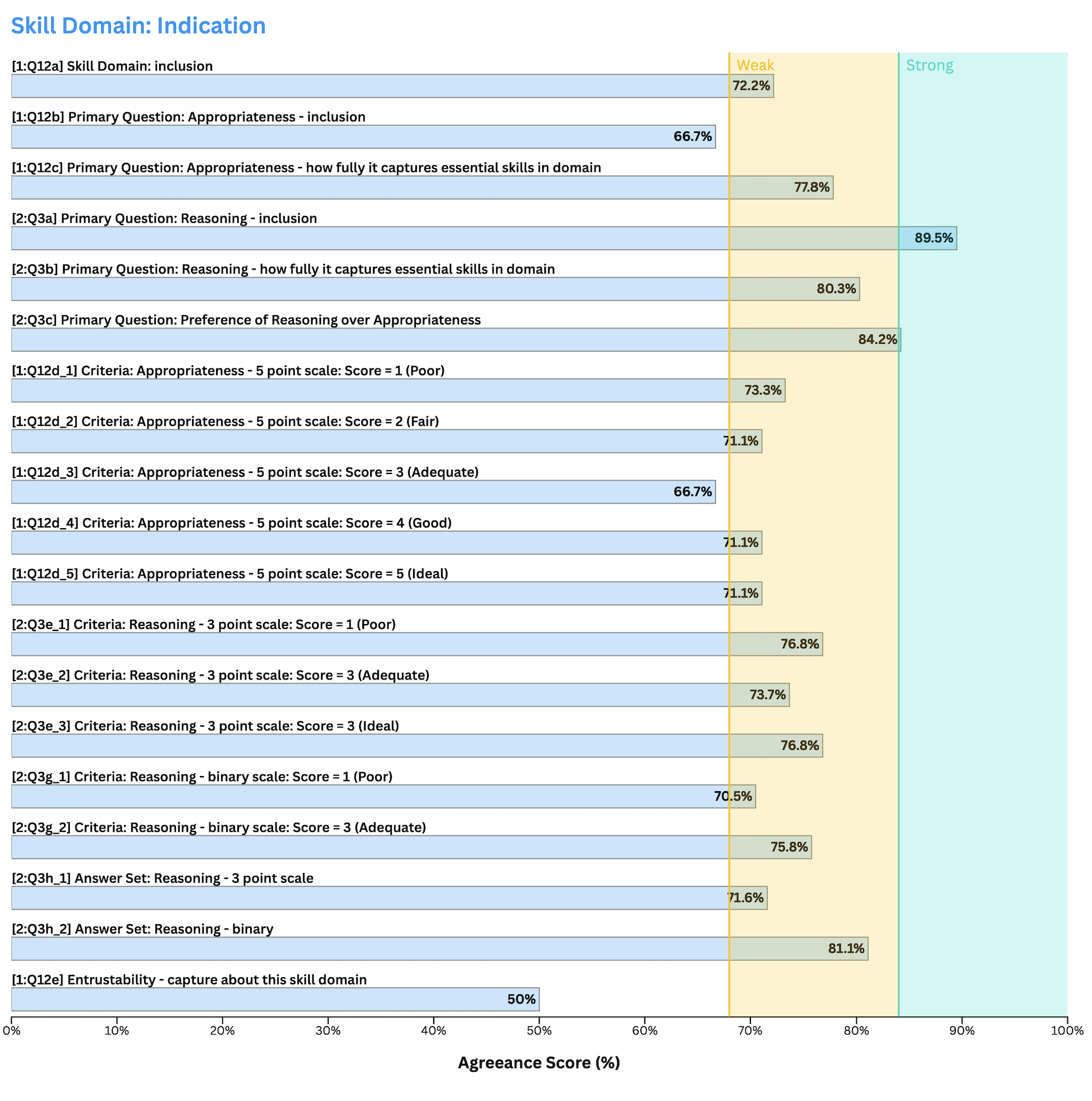


**Figure S8**. Skill domain: acquisition - Delphi result


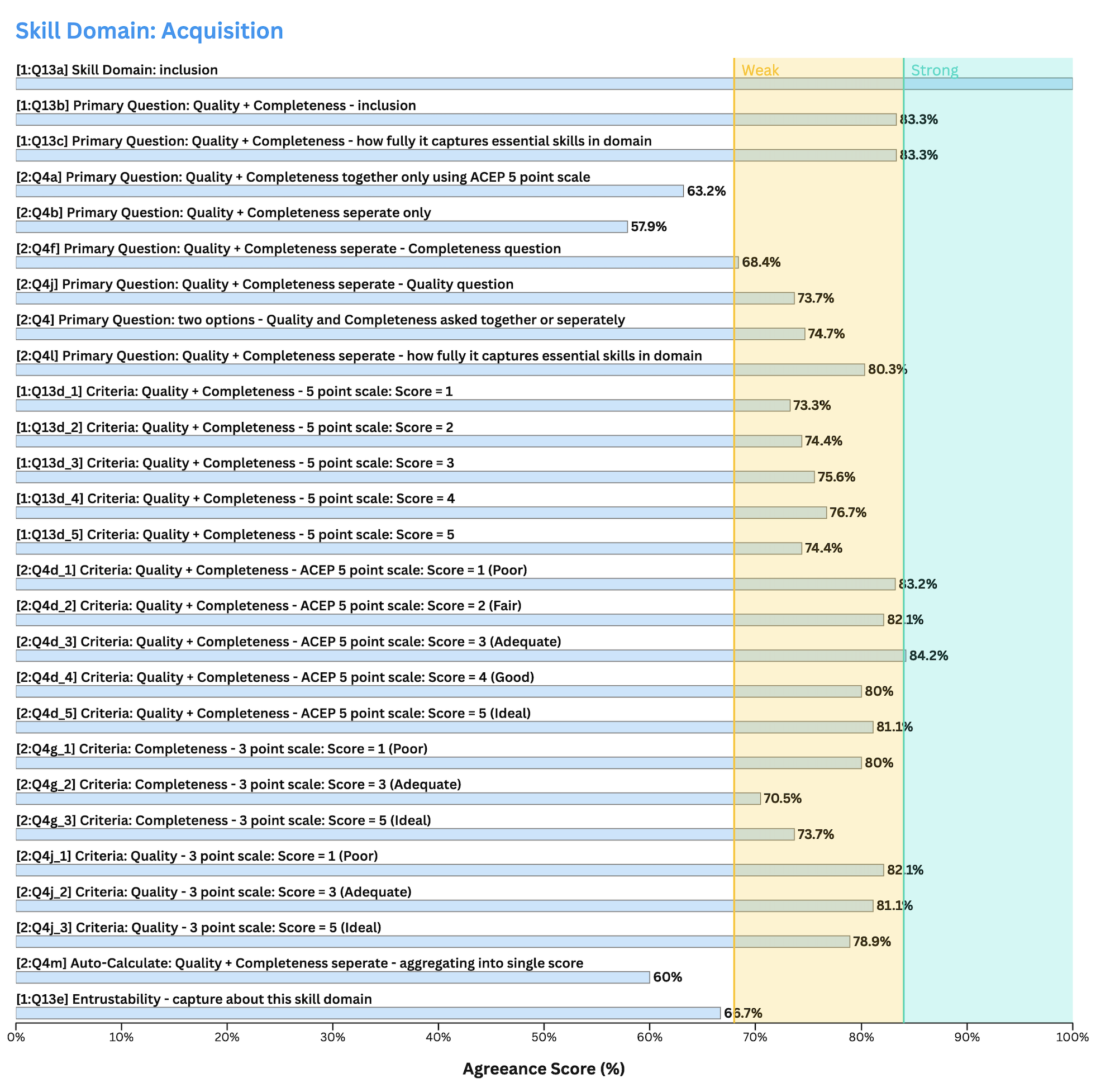


**Figure S9**. Skill domain: interpretation - Delphi result


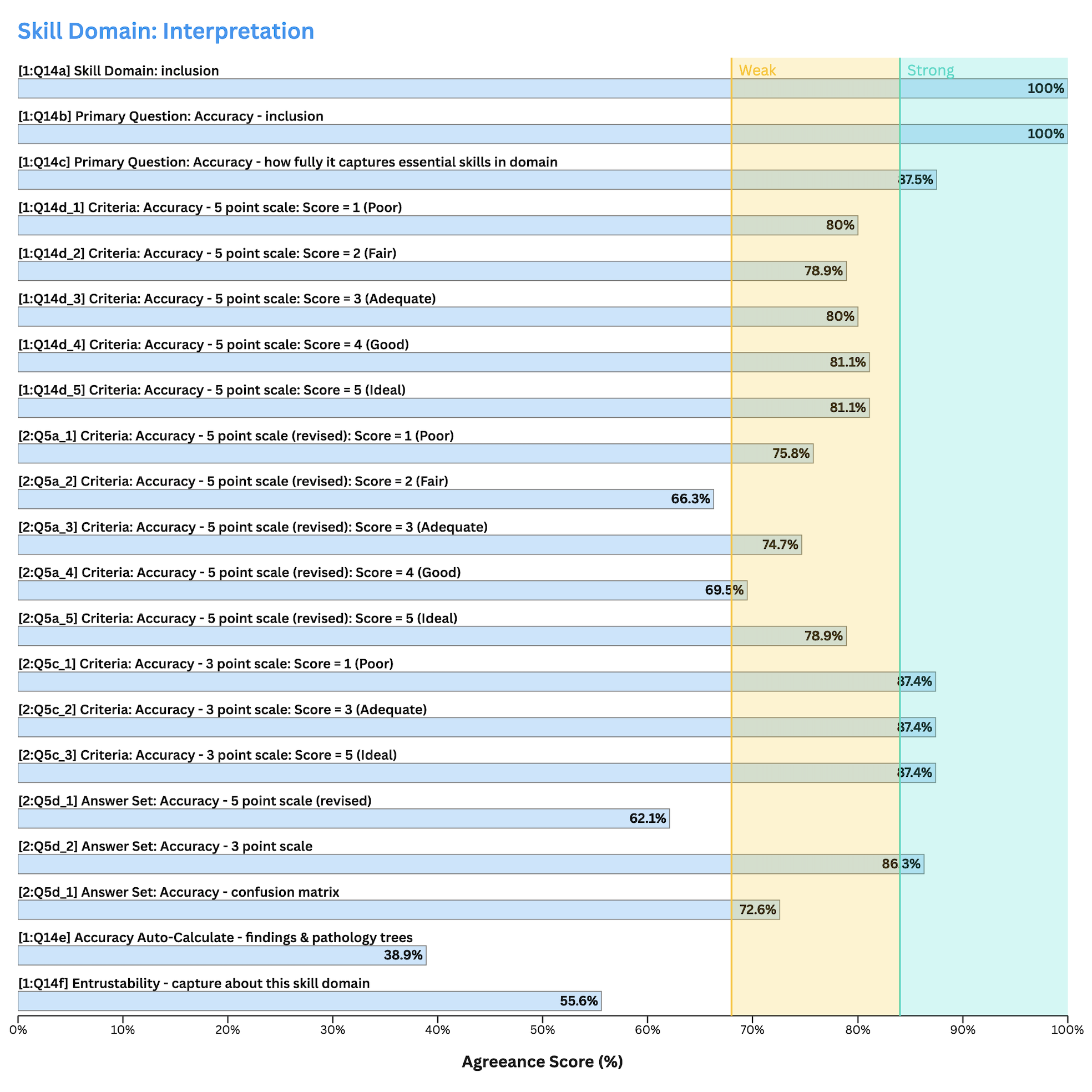


**Figure S10**. Skill domain: clinical integration - Delphi result

**
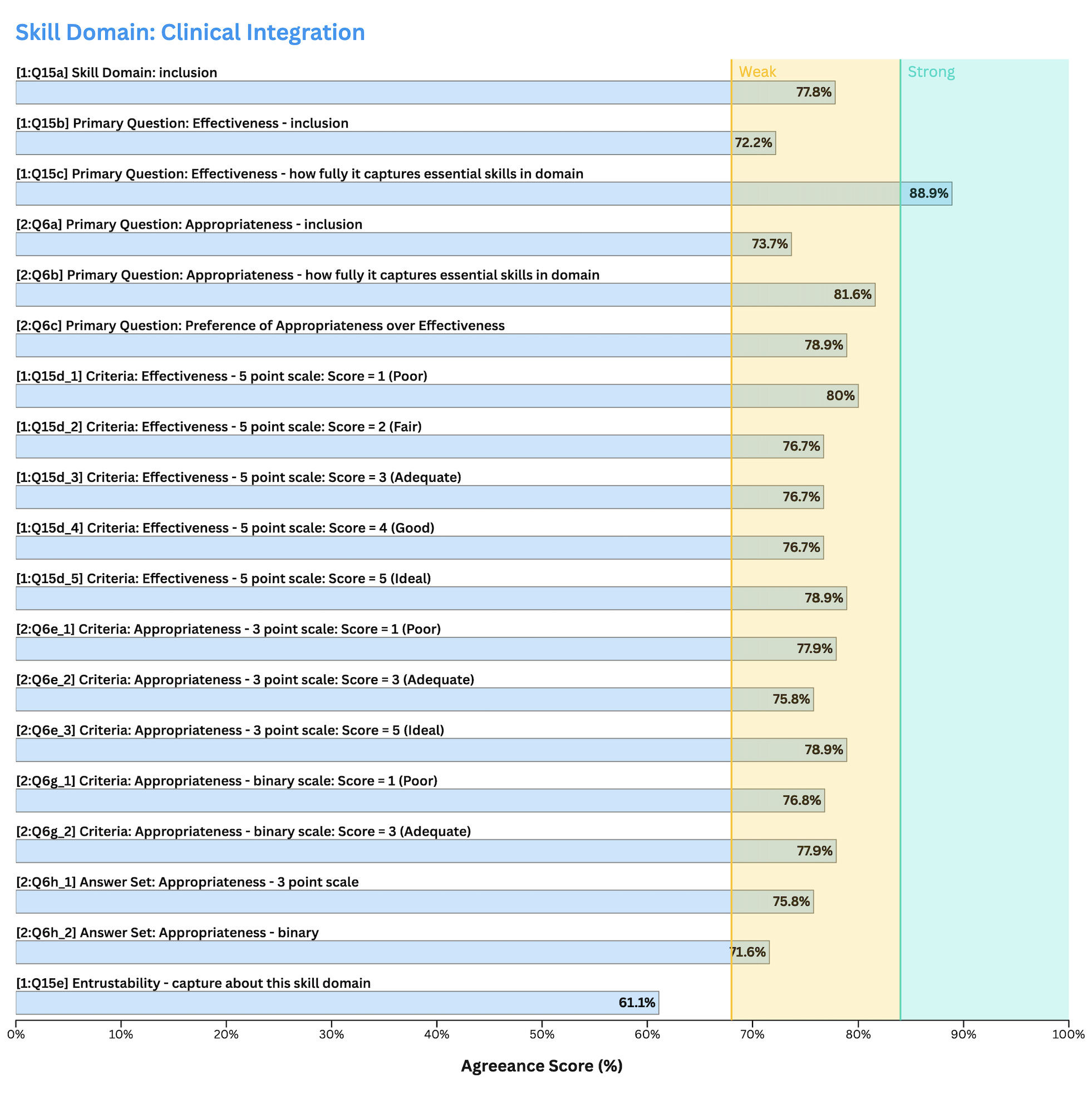
**

**Figure S11**. Skill tree: acquisition - Delphi result


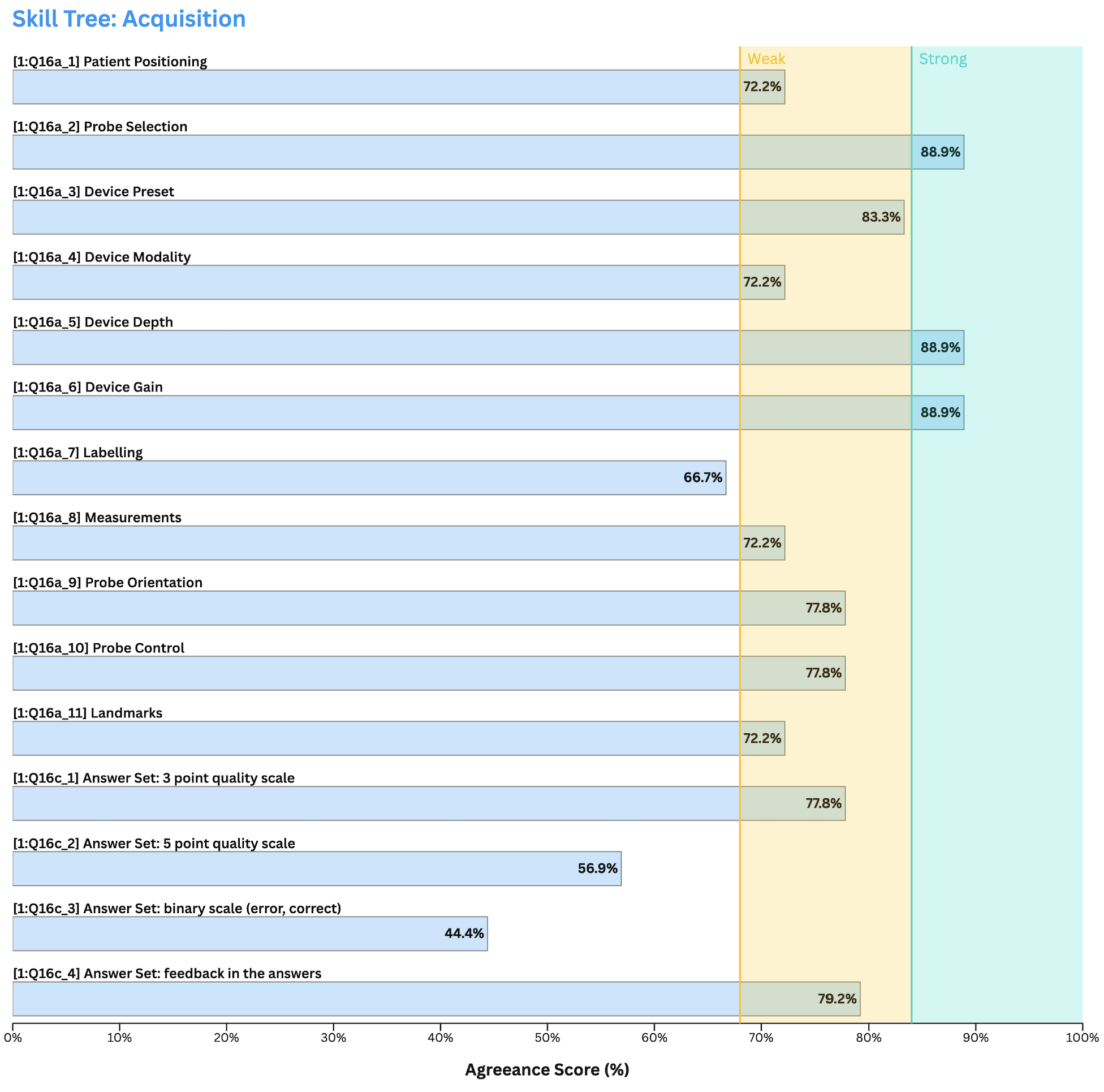


**Figure S12**. Metadata - Delphi result


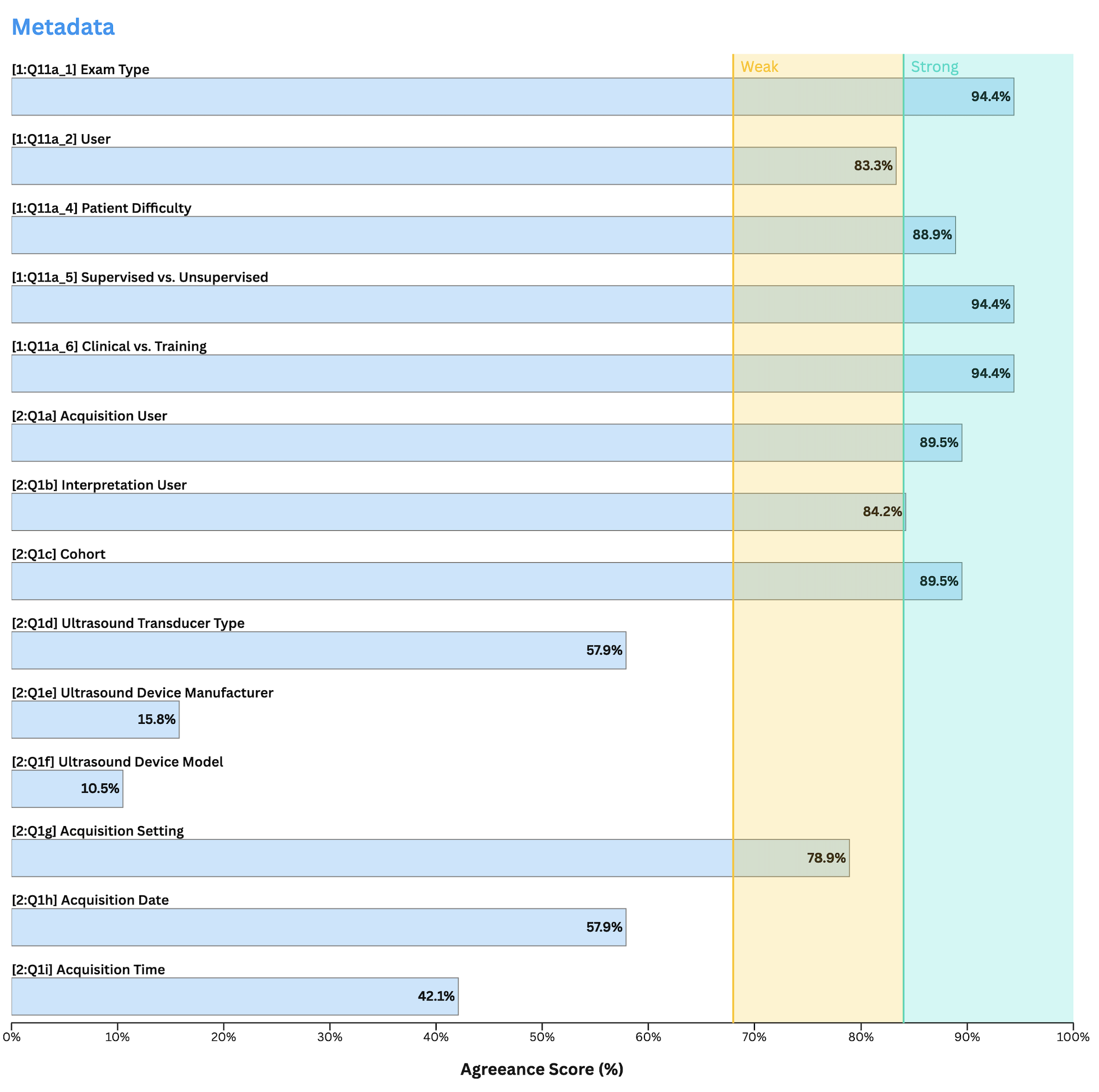


| **Table S1.** Delphi round 1: global competency domains and score | | | | | | |
| --- | --- | --- | --- | --- | --- | --- |
| **Question** | | **Agreeance** | | **Responses** | | |
| **ID** | **Text** | **Score (%)** | **Consensus** | **Include (n)** | **Neutral (n)** | **Exclude (n)** |
|  | **Experience** |  |  |  |  |  |
| 1:Q2a | Exam Counts: do you recommend this for inclusion in the framework? | 94.4 | strong | 17 | 1 | 0 |
| 1:Q3a | Exam View Counts: Do you recommend this for inclusion in the framework? | 44.4 | none | 8 | 7 | 3 |
| 1:Q4a | Exam Findings Counts: Do you recommend this for inclusion in the framework? | 72.2 | weak | 13 | 5 | 0 |
| 1:Q5a | Exam Pathology Counts: Do you recommend this for inclusion in the framework? | 66.7 | none | 12 | 5 | 1 |
|  | **Skills** |  |  |  |  |  |
| 1:Q8b | This would be auto-calculated from the scores in the Skill Domains used (i.e. indication, acquisition, interpretation, clinical integration). The reason being is the skill questions needs to at least be specific to a particular Skill Domain, otherwise they are too generic and thus would not result in accurate competency measurement. | 55.6 | none | 10 | 6 | 2 |
| 1:Q8c | If there was an alternative 5-point scale used to answer this question (1=poor, 2=fair, 3=adequate, 4=good, 5=ideal), would you recommend inclusion of the 5-point scale in the framework?  (This would be an alternative to the auto-calculation from the Skill Domains) | 61.1 | none | 11 | 3 | 4 |
|  | **Entrustability** |  |  |  |  |  |
| 1:Q9a | Do you recommend this for inclusion in the framework? | 72.2 | weak | 13 | 3 | 2 |
|  | **Competency Score** |  |  |  |  |  |
| 1:Q10a | Do you recommend this for inclusion in the framework to be used as an alternative to asking about Skill Quality and Entrustability as two separate questions? | 61.1 | none | 11 | 1 | 6 |

| **Table S2.** Delphi round 1: entrustability and competency score criteria | | | | | | | | |
| --- | --- | --- | --- | --- | --- | --- | --- | --- |
| **Question** | | **Agreeance** | | **Responses** | | |  |  |
| **ID** | **Text** | **Score (%)** | **Consensus** | **Strongly Agree (n)** | **Agree (n)** | **Neutral (n)** | **Disagree (n)** | **Strongly Disagree (n)** |
|  | **Entrustability** |  |  |  |  |  |  |  |
| 1:Q9b | How much do you agree with the Criteria? |  |  |  |  |  |  |  |
| 1:Q9b_1 | 1 = supervisor did it | 73.3 | weak | 6 | 5 | 3 | 3 | 1 |
| 1:Q9b_2 | 2 = supervisor talked through it | 71.1 | weak | 5 | 7 | 2 | 1 | 3 |
| 1:Q9b_3 | 3 = supervisor needed to prompt | 82.2 | weak | 6 | 8 | 4 | 0 | 0 |
| 1:Q9b_4 | 4 = supervisor needed to be there just in case | 77.8 | weak | 5 | 8 | 4 | 0 | 1 |
| 1:Q9b_5 | 5 = supervisor did not need to be there | 88.9 | strong | 10 | 6 | 2 | 0 | 0 |
|  | **Competency Score** |  |  |  |  |  |  |  |
| 1:Q10b | How much do you agree with the Criteria? |  |  |  |  |  |  |  |
| 1:Q10b_0 | 0 = Not attempted yet | 67.8 | none | 4 | 5 | 6 | 0 | 3 |
| 1:Q10b_1 | 1 = Requires close supervision for most of exam | 85.6 | strong | 8 | 8 | 1 | 1 | 0 |
| 1:Q10b_2 | 2 = Can complete parts of the examination independently under close supervision | 83.3 | weak | 7 | 8 | 2 | 1 | 0 |
| 1:Q10b_3 | 3 = Adequate practical skills. Can complete straightforward parts of the examination but needs close supervision for more challenging aspects or support with image interpretation | 84.4 | strong | 8 | 6 | 4 | 0 | 0 |
| 1:Q10b_4 | 4 = Good integration of practical skills and knowledge. Able to consistently complete both straightforward and more complicated parts of the examination with minimal supervisor intervention | 83.3 | weak | 7 | 7 | 4 | 0 | 0 |
| 1:Q10b_5 | 5 = Excellent integration of practical skills and knowledge. Able to consistently complete full scans to the standard of a qualified (first post) practitioner, including complicated aspects. Recognises when a second opinion is required. | 85.6 | strong | 8 | 7 | 3 | 0 | 0 |

| **Table S3.** Delphi round 1: metadata items | | | | | | |
| --- | --- | --- | --- | --- | --- | --- |
| **Question** | | **Agreeance** | | **Responses** | | |
| **ID** | **Text** | **Score (%)** | **Consensus** | **Include (n)** | **Neutral (n)** | **Exclude (n)** |
|  | Do you recommend each for inclusion in the framework? |  |  |  |  |  |
| 1:Q11a_1 | **Exam type:** (i.e. Cardiac, Lung, E-FAST, etc.) | 94.4 | strong | 17 | 1 | 0 |
| 1:Q11a_2 | **User:** who acquired and/or interpreted the ultrasound exam. | 83.3 | weak | 15 | 3 | 0 |
| 1:Q11a_4 | **Patient Difficulty:** if there were challenges performing the ultrasound exam due to patient circumstance | 88.9 | strong | 16 | 2 | 0 |
| 1:Q11a_5 | **Supervised vs. unsupervised:** whether or not the exam performed under supervision | 94.4 | strong | 17 | 1 | 0 |
| 1:Q11a_6 | **Clinical vs. training:** if the exam was performed clinically or for training purposes | 94.4 | strong | 17 | 1 | 0 |

| **Table S4.** Delphi round 1: skill domains and metrics | | | | | | |
| --- | --- | --- | --- | --- | --- | --- |
| **Question** | | **Agreeance** | | **Responses** | | |
| **ID** | **Text** | **Score (%)** | **Consensus** | **Include (n)** | **Neutral (n)** | **Exclude (n)** |
|  | **Indication**  **Metric**  Appropriateness - is the ultrasound exam chosen appropriate considering the patient's symptoms and medical history? |  |  |  |  |  |
| 1:Q12a | Do you recommend this Skill Domain for inclusion in the framework? | 72.2 | weak | 13 | 5 | 0 |
| 1:Q12b | Do you recommend the Metric for the Skill Domain for inclusion in the framework? | 66.7 | none | 12 | 4 | 2 |
| 1:Q12e | Do you recommend capturing Entrustability about this Skill Domain for inclusion in the framework? | 50.0 | none | 9 | 7 | 2 |
|  | **Acquisition**  **Metric**  Quality and Completeness - were the acquired ultrasound scans of enough completeness and quality to answer the clinical question? |  |  |  |  |  |
| 1:Q13a | Do you recommend this Skill Domain for inclusion in the framework? | 100.0 | strong | 18 | 0 | 0 |
| 1:Q13b | Do you recommend the Metric for the Skill Domain for inclusion in the framework? | 83.3 | weak | 15 | 2 | 1 |
| 1:Q13e | Do you recommend capturing Entrustability about this Skill Domain for inclusion in the framework? | 66.7 | none | 12 | 4 | 2 |
|  | **Interpretation**  **Metric**  Accuracy - were relevant findings and pathology accurately interpreted? |  |  |  |  |  |
| 1:Q14a | Do you recommend this Skill Domain for inclusion in the framework? | 100.0 | strong | 18 | 0 | 0 |
| 1:Q14b | Do you recommend the Metric for the Skill Domain for inclusion in the framework? | 100.0 | strong | 18 | 0 | 0 |
| 1:Q14f | Do you recommend capturing Entrustability about this Skill Domain for inclusion in the framework? | 55.6 | none | 10 | 7 | 1 |
|  | **Clinical Integration**  **Metric**  Effectiveness - were ultrasound findings effectively integrated into clinical decision-making and patient management? |  |  |  |  |  |
| 1:Q15a | Do you recommend this Skill Domain for inclusion in the framework? | 77.8 | weak | 14 | 3 | 1 |
| 1:Q15b | Do you recommend the Metric for the Skill Domain for inclusion in the framework? | 72.2 | weak | 13 | 4 | 1 |
| 1:Q15e | Do you recommend capturing Entrustability about this Skill Domain for inclusion in the framework? | 61.1 | none | 11 | 6 | 1 |

| **Table S5.** Delphi round 1: skill domain metric score criteria | | | | | | | | |
| --- | --- | --- | --- | --- | --- | --- | --- | --- |
| **Question** | | **Agreeance** | | **Responses** | | |  |  |
| **ID** | **Text** | **Score (%)** | **Consensus** | **Strongly Agree (n)** | **Agree (n)** | **Neutral (n)** | **Disagree (n)** | **Strongly Disagree (n)** |
|  | **Indication**  **Metric**  Appropriateness - is the ultrasound exam chosen appropriate considering the patient's symptoms and medical history? |  |  |  |  |  |  |  |
| 1:Q12d | How much do you agree with the Criteria for the Metric? |  |  |  |  |  |  |  |
| 1:Q12d_1 | 1 = Poor: Ultrasound use was inappropriate and uninformative | 73.3 | weak | 6 | 5 | 3 | 3 | 1 |
| 1:Q12d_2 | 2 = Fair: Ultrasound had limited relevance and diagnostic clarity | 71.1 | weak | 5 | 5 | 5 | 1 | 2 |
| 1:Q12d_3 | 3 = Adequate: Ultrasound was correctly performed and clinically useful | 66.7 | none | 3 | 7 | 4 | 1 | 3 |
| 1:Q12d_4 | 4 = Good: Ultrasound provided clear diagnostic value and impacted clinical decisions positively | 71.1 | weak | 5 | 6 | 3 | 2 | 2 |
| 1:Q12d_5 | 5 = Ideal: Ultrasound was exemplary, critical for diagnosis, and pivotal for patient management | 71.1 | weak | 5 | 6 | 3 | 2 | 2 |
|  | **Acquisition**  **Metric**  Quality and Completeness - were the acquired ultrasound scans of enough completeness and quality to answer the clinical question?  This can be asked for each individual scan or for the exam as a whole. |  |  |  |  |  |  |  |
| 1:Q13d | How much do you agree with the Criteria for the Metric? |  |  |  |  |  |  |  |
| 1:Q13d_1 | 1 = Poor: Images incomplete; poor ergonomic and probe use; fails diagnostic standards | 73.3 | weak | 6 | 7 | 1 | 1 | 3 |
| 1:Q13d_2 | 2 = Fair: Images have minimal diagnostic value; basic ergonomic and probe use | 74.4 | weak | 6 | 6 | 3 | 1 | 2 |
| 1:Q13d_3 | 3 = Adequate: Clinically acceptable images; meets diagnostic Criteria:; effective probe and ergonomic application | 75.6 | weak | 6 | 7 | 2 | 1 | 2 |
| 1:Q13d_4 | 4 = Good: High-quality images; advanced ergonomic and probe use; strong troubleshooting skills | 76.7 | weak | 6 | 8 | 1 | 1 | 2 |
| 1:Q13d_5 | 5 = Ideal: Exceptional images in all aspects; optimal probe and ergonomic practice; surpasses all diagnostic standards | 74.4 | weak | 6 | 7 | 1 | 2 | 2 |
|  | **Interpretation**  **Metric**  Accuracy - were relevant findings and pathology accurately interpreted?  This can be asked for each individual scan or for the exam as a whole. |  |  |  |  |  |  |  |
| 1:Q14d | How much do you agree with the Criteria for the Metric? |  |  |  |  |  |  |  |
| 1:Q14d_1 | 1 = Poor: Interpretation was inappropriate and uninformative | 80.0 | weak | 7 | 8 | 0 | 2 | 1 |
| 1:Q14d_2 | 2 = Fair: Interpretation had limited relevance and diagnostic clarity | 78.9 | weak | 6 | 7 | 3 | 2 | 0 |
| 1:Q14d_3 | 3 = Adequate: Interpretation was mostly correct and clinically useful | 80.0 | weak | 8 | 5 | 2 | 3 | 0 |
| 1:Q14d_4 | 4 = Good: Interpretation provided clear diagnostic value and impacted clinical decisions positively, although some minor inaccuracies were found | 81.1 | weak | 9 | 4 | 2 | 3 | 0 |
| 1:Q14d_5 | 5 = Ideal: Interpretation was exemplary, critical for diagnosis, and pivotal for patient management | 81.1 | weak | 9 | 4 | 2 | 3 | 0 |
|  | **Clinical Integration**  **Metric**  Effectiveness - were ultrasound findings effectively integrated into clinical decision-making and patient management? |  |  |  |  |  |  |  |
| 1:Q15d | How much do you agree with the Criteria for the Metric? |  |  |  |  |  |  |  |
| 1:Q15d_1 | 1 = Poor: Findings poorly applied; inadequate clinical integration; negatively impacts care | 80.0 | weak | 7 | 6 | 4 | 0 | 1 |
| 1:Q15d_2 | 2 = Fair: Limited application of findings; basic clinical integration; minimal impact on care | 76.7 | weak | 6 | 5 | 6 | 0 | 1 |
| 1:Q15d_3 | 3 = Adequate: Correct application of findings; solid clinical knowledge; appropriate care impact | 76.7 | weak | 5 | 6 | 6 | 1 | 0 |
| 1:Q15d_4 | 4 = Good: Effective findings application; strong clinical integration; positive care outcomes | 76.7 | weak | 6 | 5 | 5 | 2 | 0 |
| 1:Q15d_5 | 5 = Ideal: Exceptional application and integration; optimal care impact, matching expert standards | 78.9 | weak | 6 | 6 | 5 | 1 | 0 |

| **Table S6.** Delphi round 1: acquisition skill tree | | | | | | |
| --- | --- | --- | --- | --- | --- | --- |
| **Question** | | **Agreeance** | | **Responses** | | |
| **ID** | **Text** | **Score (%)** | **Consensus** | **Include (n)** | **Neutral (n)** | **Exclude (n)** |
| 1:Q16a | Specify your recommendations for inclusion of each micro skill for the Acquisition Skill Tree in the Framework |  |  |  |  |  |
| 1:Q16a_1 | **Patient positioning:** Aligning the patient for optimal imaging access. | 72.2 | weak | 13 | 4 | 1 |
| 1:Q16a_2 | **Probe selection:** Choosing the right ultrasound probe for the exam. | 88.9 | strong | 16 | 2 | 0 |
| 1:Q16a_3 | **Device preset:** Pre-configured settings for specific exam types. | 83.3 | weak | 15 | 3 | 0 |
| 1:Q16a_4 | **Device modality:** The ultrasound technique applied, e.g., B-mode, Doppler. | 72.2 | weak | 13 | 4 | 1 |
| 1:Q16a_5 | **Device depth:** Setting for ultrasound wave penetration depth. | 88.9 | strong | 16 | 2 | 0 |
| 1:Q16a_6 | **Device gain:** Adjustment of image brightness. | 88.9 | strong | 16 | 2 | 0 |
| 1:Q16a_7 | **Labeling:** Annotating images with identifiers or findings. | 66.7 | none | 12 | 5 | 1 |
| 1:Q16a_8 | **Measurements:** Quantifying anatomical sizes or pathologies on images. | 72.2 | weak | 13 | 5 | 0 |
| 1:Q16a_9 | **Probe orientation:** Aligning the probe with anatomical directions. | 77.8 | weak | 14 | 4 | 0 |
| 1:Q16a_10 | **Probe control:** Handling the probe for optimal image capture. | 77.8 | weak | 14 | 3 | 1 |
| 1:Q16a_11 | **Landmarks:** Recognizable features used for navigation and interpretation. | 72.2 | weak | 13 | 4 | 1 |

| **Table S7.** Delphi round 1: acquisition skill tree - answer sets | | | | | | |  |
| --- | --- | --- | --- | --- | --- | --- | --- |
| **Question** | | **Agreeance** | | **Responses** | | |  |
| **ID** | **Text** | **Score (%)** | **Consensus** | **1 (Most useful)** | **2** | **3** | **4 (Least useful)** |
| 1:Q16c_1 | Three Point Quality Scale | 77.8 | weak | 8 | 4 | 6 | 0 |
| 1:Q16c_2 | Five Point Quality Scale | 56.9 | none | 2 | 6 | 5 | 5 |
| 1:Q16c_3 | Binary | 44.4 | none | 1 | 4 | 3 | 10 |
| 1:Q16c_4 | Feedback in the answers | 79.2 | weak | 9 | 5 | 2 | 2 |

| **Table S8.** Delphi round 2: metadata new items | | | | | | |
| --- | --- | --- | --- | --- | --- | --- |
| **Question** | | **Agreeance** | | **Responses** | | |
| **ID** | **Text** | **Score (%)** | **Consensus** | **Include (n)** | **Neutral (n)** | **Exclude (n)** |
| 2:Q1 | Do you recommend each for inclusion in the framework? |  |  |  |  |  |
| 2:Q1a | **Acquisition User:** who acquired the ultrasound scans | 89.5 | strong | 17 | 2 | 0 |
| 2:Q1b | **Interpretation User:** who interpreted the ultrasound scans (if applicable - in most cases it's the same user who acquired the scan, but some ultrasound users only acquire or interpret) | 84.2 | strong | 16 | 3 | 0 |
| 2:Q1c | **Cohort:** which group or cohort the user is associated with - enables generation of competency curves across cohorts. | 89.5 | strong | 17 | 2 | 0 |
| 2:Q1d | **Ultrasound Transducer Type:** i.e. phased, linear, curved linear | 57.9 | none | 11 | 5 | 3 |
| 2:Q1e | **Ultrasound Device Manufacturer:** i.e. GE, butterfly, phillips | 15.8 | none | 3 | 9 | 7 |
| 2:Q1f | **Ultrasound Device Model:** specific model from the manufacturer i.e. (Butterfly iQ+, Vscan Air, Lumify) | 10.5 | none | 2 | 10 | 7 |
| 2:Q1g | **Acquisition Setting:** i.e. ICU, ED, outpatient, etc. | 78.9 | weak | 15 | 3 | 1 |
| 2:Q1h | **Acquisition Date:** specific date the ultrasound was performed on | 57.9 | none | 11 | 6 | 2 |
| 2:Q1i | **Acquisition Time:** specific time ultrasound was performed at | 42.1 | none | 8 | 8 | 3 |

| **Table S9.** Delphi round 2: skill domain - indication - new metric | | | | | | |  |  |
| --- | --- | --- | --- | --- | --- | --- | --- | --- |
| **Question** | | **Agreeance** | | **Responses** | | |  |  |
| **ID** | **Text** | **Score (%)** | **Consensus** | **Include (n)** | **Neutral (n)** | **Exclude (n)** | **Yes (n)** | **No (n)** |
|  | **Indication**  **New Metric:**  Reasoning - can the practitioner articulate a focused clinical question that ultrasound can address for this patient? |  |  |  |  |  |  |  |
| 2:Q3a | Do you recommend the New Metric for the Skill Domain for inclusion in the framework? | 89.5 | strong | 17 | 2 | 0 |  |  |
| 2:Q3c | Do you prefer the New Metric (Reasoning) over the Previous Metric (Appropriateness)? | 84.2 | strong |  |  |  | 16 | 3 |

| **Table S10.** Delphi round 2: skill domain - indication - new metric score criteria | | | | | | | |  |
| --- | --- | --- | --- | --- | --- | --- | --- | --- |
| **Question** | | **Agreeance** | | **Responses** | | | | |
| **ID** | **Text** | **Score (%)** | **Consensus** | **Strongly Agree (n)** | **Agree (n)** | **Neutral (n)** | **Disagree (n)** | **Strongly Disagree (n)** |
|  | **Indication**  **New Metric:**  Reasoning - can the practitioner articulate a focused clinical question that ultrasound can address for this patient? |  |  |  |  |  |  |  |
| 2:Q3e | How much do you agree with the criteria to answer the New Metric? |  |  |  |  |  |  |  |
| 2:Q3e_1 | 1 = Poor: no relevant clinical question formulated | 76.8 | weak | 5 | 8 | 5 | 0 | 1 |
| 2:Q3e_2 | 3 = Adequate: relevant clinical question formulated, but it lacks specificity to the patient's condition | 73.7 | weak | 3 | 10 | 4 | 1 | 1 |
| 2:Q3e_3 | 5 = Ideal: relevant clinical question formulated, and is specific to the patient's condition | 76.8 | weak | 7 | 5 | 5 | 1 | 1 |
| 2:Q3g | Alternatively, this could be answered with binary criteria. How much do you agree with the binary criteria to answer the New Metric? |  |  |  |  |  |  |  |
| 2:Q3g_1 | Poor: does not provide a relevant clinical question for ultrasound evaluation, or the question is unclear or lacks specificity. | 70.5 | weak | 6 | 4 | 4 | 4 | 1 |
| 2:Q3g_2 | Adequate: relevant clinical question formulated | 75.8 | weak | 7 | 6 | 2 | 3 | 1 |
| 2:Q3h | Which allowable answer sets do you recommend including in the Framework for the New Metric - Reasoning?  Since there is matching criteria both allowable answer sets can be included so ultrasound programs can pick their level of granularity. |  |  |  |  |  |  |  |
| 2:Q3h_1 | 3-point scale | 71.6 | weak | 4 | 8 | 4 | 1 | 2 |
| 2:Q3h_2 | Binary | 81.1 | weak | 10 | 3 | 3 | 3 | 0 |

| **Table S11.** Delphi round 2: skill domain - acquisition - new metrics | | | | | | |
| --- | --- | --- | --- | --- | --- | --- |
| **Question** | | **Agreeance** | | **Responses** | | |
| **ID** | **Text** | **Score (%)** | **Consensus** | **Include (n)** | **Neutral (n)** | **Exclude (n)** |
|  | **Acquisition**  **Option 1) Single metric:**  ACEP 5-point grading scale  Completeness is part of the criteria (score >2 means complete)  **Option 2) Two metrics:**  Completeness: Were all required ultrasound scan views captured to answer the clinical question?  Quality: Were the ultrasound scans of sufficient quality to allow accurate interpretation and answer the clinical question?  Quality and completeness aren't always congruent - this allows for more accurate measurement of acquisition skills in scenarios where the scans are high quality but not complete, or complete but not high quality. This can be asked for each individual scan or for the exam as a whole. |  |  |  |  |  |
| 2:Q4a | Do you recommend only Option 1 (ACEP 5-point grading scale) for inclusion in the framework within the Acquisition Skill Domain? | 63.2 | none | 12 | 5 | 2 |
| 2:Q4b | Do you recommend only Option 2 (Two metrics - completeness and quality) for inclusion in the framework within the Acquisition Skill Domain? | 57.9 | none | 11 | 5 | 3 |
| 2:Q4f | Do you recommend Completeness for option 2 (above) to be included in the framework for the acquisition skill domain? | 68.4 | weak | 13 | 4 | 2 |
| 2:Q4i | Do you recommend Quality for option 2 (above) to be included in the framework for the acquisition skill domain? | 73.7 | weak | 14 | 4 | 1 |

| **Table S12.** Delphi round 2: skill domain - acquisition - revised metrics and score criteria | | | | | | | |  |
| --- | --- | --- | --- | --- | --- | --- | --- | --- |
| **Question** | | **Agreeance** | | **Responses** | | | | |
| **ID** | **Text** | **Score (%)** | **Consensus** | **Strongly Agree (n)** | **Agree (n)** | **Neutral (n)** | **Disagree (n)** | **Strongly Disagree (n)** |
|  | **Skill Domain** - Acquisition  **Option 1) Single metric:**  ACEP 5-point grading scale  Completeness is part of the criteria (score >2 means complete)  **Option 2) Two metrics:**  Completeness: Were all required ultrasound scan views captured to answer the clinical question?  Quality: Were the ultrasound scans of sufficient quality to allow accurate interpretation and answer the clinical question?  Quality and completeness aren't always congruent - this allows for more accurate measurement of acquisition skills in scenarios where the scans are high quality but not complete, or complete but not high quality. This can be asked for each individual scan or for the exam as a whole. |  |  |  |  |  |  |  |
| 2:Q4 | Do you recommend including both options in the framework where an ultrasound program can pick one of them?  A lot of ultrasound programs in North America are already using the ACEP scale (option 1) so making it an option would increase the adoptability of this framework.  It has been noted in the feedback from round 1 that there is a desire to split this out into 2 questions (option 2) for more accuracy in measuring acquisition competency - this could be more attractive to ultrasound programs who aren't already using the ACEP scale (option 1). | 74.7 | weak | 5 | 7 | 5 | 1 | 1 |
| 2:Q4d | How much do you agree with the criteria for the ACEP 5-point grading scale? |  |  |  |  |  |  |  |
| 2:Q4d_1 | 1 = Poor: no recognizable structures | 83.2 | weak | 8 | 9 | 0 | 1 | 1 |
| 2:Q4d_2 | 2 = Fair: minimally recognizable structures but insufficient for diagnosis | 82.1 | weak | 8 | 8 | 1 | 1 | 1 |
| 2:Q4d_3 | 3 = Adequate: minimal criteria met for diagnosis, recognizable structures but with some technical or other flaws | 84.2 | strong | 7 | 10 | 1 | 1 | 0 |
| 2:Q4d_4 | 4 = Good: minimal criteria met for diagnosis, all structures imaged well | 80.0 | weak | 7 | 8 | 2 | 1 | 1 |
| 2:Q4d_5 | 5 = Ideal: minimal criteria met for diagnosis, all structures imaged with excellent image quality | 81.1 | weak | 8 | 7 | 2 | 1 | 1 |
| 2:Q4g | How much do you agree with the criteria for Metric 1 - Completeness? |  |  |  |  |  |  |  |
| 2:Q4g_1 | 1 = Poor: one or more required scan views missing | 80.0 | weak | 6 | 8 | 4 | 1 | 0 |
| 2:Q4g_2 | 3 = Adequate: required scan views are complete, one or more optional scan views missing | 70.5 | weak | 4 | 8 | 3 | 2 | 2 |
| 2:Q4g_3 | 5 = Ideal: required and optional scan views are complete | 73.7 | weak | 5 | 7 | 4 | 2 | 1 |
| 2:Q4j | How much do you agree with the criteria for Metric 2 - Quality? |  |  |  |  |  |  |  |
| 2:Q4j_1 | 1 = Poor: scans do not allow for accurate interpretation | 82.1 | weak | 7 | 10 | 0 | 1 | 1 |
| 2:Q4j_2 | 3 = Adequate: scans allow for accurate interpretation, but scan quality could be improved | 81.1 | weak | 6 | 11 | 0 | 1 | 1 |
| 2:Q4j_3 | 5 = Ideal: scans allow for accurate interpretation with excellent diagnostic scan quality | 78.9 | weak | 5 | 11 | 1 | 1 | 1 |

| **Table S13.** Delphi round 2: skill domain - interpretation - metric revised criteria | | | | | | | | |
| --- | --- | --- | --- | --- | --- | --- | --- | --- |
| **Question** | | **Agreeance** | | **Responses** | | | | |
| **ID** | **Text** | **Score (%)** | **Consensus** | **Strongly Agree (n)** | **Agree (n)** | **Neutral (n)** | **Disagree (n)** | **Strongly Disagree (n)** |
|  | **Interpretation**  **Metric**  Accuracy - were relevant findings and pathology accurately interpreted?  This can be asked for each individual scan or for the exam as a whole. |  |  |  |  |  |  |  |
| 2:Q5a | How much do you agree with the 5-point scale criteria to answer the Metric? |  |  |  |  |  |  |  |
| 2:Q5a_1 | 1 = Poor: Multiple major inaccuracies. | 75.8 | weak | 5 | 8 | 3 | 3 | 0 |
| 2:Q5a_2 | 2 = Fair: Single major inaccuracy. | 66.3 | none | 4 | 5 | 3 | 7 | 0 |
| 2:Q5a_3 | 3 = Adequate: Multiple minor inaccuracies - no major inaccuracies | 74.7 | weak | 6 | 6 | 3 | 4 | 0 |
| 2:Q5a_4 | 4 = Good: Single minor inaccuracy - no major inaccuracies | 69.5 | weak | 5 | 5 | 3 | 6 | 0 |
| 2:Q5a_5 | 5 = Ideal: No minor or major inaccuracies | 78.9 | weak | 7 | 7 | 2 | 3 | 0 |
| 2:Q5c | Alternatively, this could be answered with a 3-point scale. How much do you agree with the binary criteria to answer the Metric? |  |  |  |  |  |  |  |
| 2:Q5c_1 | 1 = Poor: Major inaccuracies. | 87.4 | strong | 11 | 5 | 2 | 1 | 0 |
| 2:Q5c_2 | 3 = Adequate: Minor inaccuracies only. | 87.4 | strong | 11 | 5 | 2 | 1 | 0 |
| 2:Q5c_3 | 5 = Ideal: No minor or major inaccuracies | 87.4 | strong | 11 | 5 | 2 | 1 | 0 |
| 2:Q5d | Which allowable answer sets do you recommend including in the Framework for answering the Metric - Accuracy?  Since there are matching criteria between 3 and 5-point scales both allowable answer sets can be included so ultrasound programs can pick their level of granularity.  The confusion matrix could be used in addition to the 3 or 5-point scales or by itself. |  |  |  |  |  |  |  |
| 2:Q5d_1 | 5-point scale | 62.1 | none | 1 | 7 | 5 | 5 | 1 |
| 2:Q5d_2 | 3-point scale | 86.3 | strong | 9 | 7 | 3 | 0 | 0 |
| 2:Q5d_1 | Confusion Matrix | 72.6 | weak | 7 | 5 | 2 | 3 | 2 |

| **Table S14.** Delphi round 2: skill domain - clinical integration - new metric | | | | | | |  |  |
| --- | --- | --- | --- | --- | --- | --- | --- | --- |
| **Question** | | **Agreeance** | | **Responses** | | |  |  |
| **ID** | **Text** | **Score (%)** | **Consensus** | **Include (n)** | **Neutral (n)** | **Exclude (n)** | **Yes (n)** | **No (n)** |
|  | **Clinical Integration**  **New Metric**  Appropriateness - were ultrasound findings appropriately integrated into clinical decision-making? |  |  |  |  |  |  |  |
| 2:Q6a | Do you recommend the New Metric for the Skill Domain for inclusion in the framework? | 73.7 | weak | 14 | 3 | 2 |  |  |
| 2:Q6c | Do you prefer the New Metric (Appropriateness) over the Previous Metric (Effectiveness)? | 78.9 | weak |  |  |  | 15 | 4 |

| **Table S15.** Delphi round 2: skill domain - clinical integration - revised metric score criteria | | | | | | | | |
| --- | --- | --- | --- | --- | --- | --- | --- | --- |
| **Question** | | **Agreeance** | | **Responses** | | | | |
| **ID** | **Text** | **Score (%)** | **Consensus** | **Strongly Agree (n)** | **Agree (n)** | **Neutral (n)** | **Disagree (n)** | **Strongly Disagree (n)** |
|  | **Clinical Integration**  **New Metric**  Appropriateness - were ultrasound findings appropriately integrated into clinical decision-making? |  |  |  |  |  |  |  |
| 2:Q6e | How much do you agree with the criteria to answer the New Metric? |  |  |  |  |  |  |  |
| 2:Q6e_1 | 1 = Poor: Fails to appropriately apply ultrasound findings to clinical decisions | 77.9 | weak | 5 | 10 | 2 | 1 | 1 |
| 2:Q6e_2 | 3 = Adequate: Applies ultrasound findings appropriately but may overlook subtle details or additional relevant information | 75.8 | weak | 5 | 8 | 4 | 1 | 1 |
| 2:Q6e_3 | 5 = Ideal: Appropriately incorporates all ultrasound findings into clinical decisions without errors or omissions | 78.9 | weak | 6 | 9 | 2 | 1 | 1 |
| 2:Q6g | Alternatively, this could be answered with binary criteria. How much do you agree with the binary criteria to answer the New Metric? |  |  |  |  |  |  |  |
| 2:Q6g_1 | Poor: Fails to appropriately apply ultrasound findings to clinical decisions | 76.8 | weak | 5 | 9 | 2 | 3 | 0 |
| 2:Q6g_2 | Adequate: Applies ultrasound findings appropriately | 77.9 | weak | 6 | 8 | 2 | 3 | 0 |
| 2:Q6h | Which allowable answer sets do you recommend including in the Framework for the New Metric - Appropriateness?  Since there is matching criteria both allowable answer sets can be included so ultrasound programs can pick their level of granularity. |  |  |  |  |  |  |  |
| 2:Q6h_1 | 3-point scale | 75.8 | weak | 7 | 5 | 3 | 4 | 0 |
| 2:Q6h_2 | Binary | 71.6 | weak | 5 | 5 | 6 | 2 | 1 |

| **Table S16.** Delphi round 2: competency score manual input | | | | | | |
| --- | --- | --- | --- | --- | --- | --- |
| **Question** | | **Agreeance** | | **Responses** | | |
| **ID** | **Text** | **Score (%)** | **Consensus** | **Include (n)** | **Neutral (n)** | **Exclude (n)** |
| 2:Q9d | For ultrasound programs with limited supervisor/expert capacity - do you recommend the manual input of competency score for inclusion in the framework to be used as an alternative option to asking about Skill Domains and Entrustability separately?  For example, the ultrasound program could start with assessments in this fashion until they have more capacity, then they could switch to asking about Skill Domains and Entrustability when ready. | 31.6 | none | 6 | 6 | 7 |

| **Table 24.** Delphi round 2: competency score auto-calculation | | | | | | | | |
| --- | --- | --- | --- | --- | --- | --- | --- | --- |
| **Question** | | **Agreeance** | | **Responses** | | | | |
| **ID** | **Text** | **Score (%)** | **Consensus** | **Strongly Agree (n)** | **Agree (n)** | **Neutral (n)** | **Disagree (n)** | **Strongly Disagree (n)** |
| 2:Q9 | Do you recommend including an auto-calculated competency score in the framework?  This is only asking about the concept of a singular competency metric that's calculated, not how it's calculated.  This aggregated score could be useful in several ways:  1) gives ultrasound programs a single metric to measure competency for each exam type (as opposed to 1 metric per domain + entrustability metric). This still allows ultrasound programs to look at the individual skill domain metrics and entrustability to see where the competency gaps are  2) allows more trends and insights to be identified in larger multi-program competency datasets | 67.4 | none | 3 | 7 | 4 | 4 | 1 |
| 2:Q9a | Do you recommend auto-calculating the competency score? | 66.3 | none | 3 | 7 | 3 | 5 | 1 |
| 2:Q9b | The following is modified criteria based on round 1 feedback. If the competency score was auto-calculated as described above, how much do you agree with the new criteria (it would be used as thresholds to describe competency at different scores)? |  |  |  |  |  |  |  |
| 2:Q9b_1 | 1 = Beginner = Requires direct supervision to perform exam | 78.9 | weak | 6 | 9 | 1 | 3 | 0 |
| 2:Q9b_2 | 2 = Limited = Performs one or more tasks independently, the rest require direct supervision | 73.7 | weak | 4 | 9 | 2 | 4 | 0 |
| 2:Q9b_3 | 3 = Adequate = Adequate skills. Performs routine exams independently; needs supervision support for complex aspects | 74.7 | weak | 4 | 10 | 1 | 4 | 0 |
| 2:Q9b_4 | 4 = Good = Good skills. Performs both routine and complex exams independently with minimal supervision | 73.7 | weak | 4 | 9 | 2 | 4 | 0 |
| 2:Q9b_5 | 5 = Ideal = Ideal skills. Performs full exams independently to practitioner standards; recognizes when to seek assistance | 78.9 | weak | 6 | 9 | 1 | 3 | 0 |

| **Table S17.** Delphi round 2: skill score auto-calculation pt.1 | | | | | | |
| --- | --- | --- | --- | --- | --- | --- |
| **Question** | | **Agreeance** | | **Responses** | | |
| **ID** | **Text** | **Score (%)** | **Consensus** | **Include (n)** | **Neutral (n)** | **Exclude (n)** |
| 2:Q7 | Aggregated Skill Domains Score: This would be auto-calculated from the scores in the Skill Domains used (i.e. indication, acquisition, interpretation, clinical integration).  Do you recommend the Aggregated Skill Domains Score for inclusion in the framework? | 63.2 | none | 12 | 5 | 2 |

| **Table S18.** Delphi round 2: skill score auto-calculation pt.2 | | | | | | | | |
| --- | --- | --- | --- | --- | --- | --- | --- | --- |
| **Question** | | **Agreeance** | | **Responses** | | | | |
| **ID** | **Text** | **Score (%)** | **Consensus** | **Strongly Agree (n)** | **Agree (n)** | **Neutral (n)** | **Disagree (n)** | **Strongly Disagree (n)** |
|  | Aggregated Skill Domains Score: This would be auto-calculated from the scores in the Skill Domains used (i.e. indication, acquisition, interpretation, clinical integration).  The calculation method would be:  Normalize each Skill Domain score to a 1-5 scale (i.e. if a 1-3 scale is used for a domain convert it into a 1-5 scale)  Aggregated Skill Score = Minimum score across all Skill Domains  The minimum score is used instead of an average score to account for scenarios like an acquisition score of 5/5 and an interpretation score of 1/5.  If both skills are required for proper clinical usage, then both need to be at least 3/5 (adequate). |  |  |  |  |  |  |  |
| 2:Q7b | Do you recommend the calculation method for the Aggregated Skill Domains Score?  (see calculation description above) | 63.2 | none | 1 | 7 | 7 | 2 | 2 |
| 2:Q7d | Thresholds can be defined to indicate varying levels of readiness based on the Aggregated Skill Domains Score.  How much do you agree with the thresholds for the Aggregated Skill Domains Score? |  |  |  |  |  |  |  |
| 2:Q7d_1 | Less than 3 (not adequate): not ready for clinical usage | 74.7 | weak | 5 | 7 | 5 | 1 | 1 |
| 2:Q7d_2 | Greater than or equal to 3 (adequate): ready for clinical usage | 75.8 | weak | 3 | 10 | 5 | 1 | 0 |
| 2:Q7d_3 | Greater than 4 (good): ready to train others | 68.4 | weak | 3 | 6 | 7 | 2 | 1 |
| 2:Q7d_4 | 5 (ideal): ready to train others (as an alternative to threshold of 4 - good) | 68.4 | weak | 6 | 2 | 7 | 2 | 2 |

| **Table S19.** Delphi round 2: entrustability revised | | | | | | | | |
| --- | --- | --- | --- | --- | --- | --- | --- | --- |
| **Question** | | **Agreeance** | | **Responses** | | | | |
| **ID** | **Text** | **Score (%)** | **Consensus** | **Strongly Agree (n)** | **Agree (n)** | **Neutral (n)** | **Disagree (n)** | **Strongly Disagree (n)** |
| 2:Q8a | Knowing that the following criteria is from the Modified Ottawa Entrustability Score (page 9 in link), how much do you agree with the criteria?  Note: for certain assessment scenarios where the supervisor cannot intervene (i.e. OSCEs), these can also be answered hypothetically based on what the supervisor would have needed to do in a clinical environment (instead of what they did). |  |  |  |  |  |  |  |
| 2:Q8a_1 | 1 = supervisor did it: trainee required complete guidance or was unprepared; supervisor had to do most of the work | 80.0 | weak | 9 | 4 | 4 | 1 | 1 |
| 2:Q8a_2 | 2 = supervisor talked through it: trainee was able to perform some tasks but required repeated directions | 83.2 | weak | 9 | 5 | 4 | 1 | 0 |
| 2:Q8a_3 | 3 = supervisor needed to prompt: trainee demonstrated some independence and only required intermittent prompting | 84.2 | strong | 9 | 6 | 3 | 1 | 0 |
| 2:Q8a_4 | 4 = supervisor needed to be there just in case: trainee functioned fairly independently and only needed assistance with nuances or complex situations | 85.3 | strong | 10 | 5 | 3 | 1 | 0 |
| 2:Q8a_5 | 5 = supervisor did not need to be there | 88.4 | strong | 11 | 5 | 3 | 0 | 0 |
| 2:Q8b | The Modified Ottawa Entrustability Score does not currently contain single word descriptors for each score. To make this scale more intuitive, how much do agree with the following single word descriptors for each score? |  |  |  |  |  |  |  |
| 2:Q8b_1 | 1 = Dependent = supervisor did it: trainee required complete guidance or was unprepared; supervisor had to do most of the work | 81.1 | weak | 8 | 7 | 2 | 1 | 1 |
| 2:Q8b_2 | 2 = Guided = supervisor talked through it: trainee was able to perform some tasks but required repeated directions | 83.2 | weak | 7 | 9 | 2 | 1 | 0 |
| 2:Q8b_3 | 3 = Prompted = supervisor needed to prompt: trainee demonstrated some independence and only required intermittent prompting | 84.2 | strong | 7 | 10 | 1 | 1 | 0 |
| 2:Q8b_4 | 4 = Monitored = supervisor needed to be there just in case: trainee functioned fairly independently and only needed assistance with nuances or complex situations | 84.2 | strong | 7 | 10 | 1 | 1 | 0 |
| 2:Q8b_5 | 5 = Independent = supervisor did not need to be there | 90.5 | strong | 10 | 9 | 0 | 0 | 0 |
| 2:Q8d | Which allowable answer sets do you recommend including in the Framework?  Since there is matching criteria all allowable answer sets can be included so ultrasound programs can pick their level of granularity, and the datasets can still be compared across programs. |  |  |  |  |  |  |  |
| 2:Q8d_1 | 5-point scale | 75.8 | weak | 6 | 8 | 1 | 3 | 1 |
| 2:Q8d_2 | 3-point scale | 71.6 | weak | 4 | 7 | 4 | 4 | 0 |
| 2:Q8d_3 | Binary scale | 49.5 | none | 2 | 2 | 2 | 10 | 3 |
